# Fossil biogeography: a new model to infer dispersal, extinction and sampling from palaeontological data

**DOI:** 10.1098/rstb.2015.0225

**Published:** 2016-04-05

**Authors:** Daniele Silvestro, Alexander Zizka, Christine D. Bacon, Borja Cascales-Miñana, Nicolas Salamin, Alexandre Antonelli

**Affiliations:** 1 Department of Biological and Environmental Sciences, University of Gothenburg, Carl Skottsbergs gata 22B, Gothenburg 413 19, Sweden; 2 Department of Ecology and Evolution, University of Lausanne, 1015 Lausanne, Switzerland; 3 Swiss Institute of Bioinformatics, Quartier Sorge, 1015 Lausanne, Switzerland; 4 Department of Geology, University of Liege, 4000 Sart Tilman, Liege, Belgium; 5 Gothenburg Botanical Garden, Carl Skottsbergs gata 22A, Gothenburg 413 19, Sweden

**Keywords:** dispersal, extinction, incomplete fossil sampling, biogeographic trends, macroevolution

## Abstract

Methods in historical biogeography have revolutionized our ability to infer the evolution of ancestral geographical ranges from phylogenies of extant taxa, the rates of dispersals, and biotic connectivity among areas. However, extant taxa are likely to provide limited and potentially biased information about past biogeographic processes, due to extinction, asymmetrical dispersals and variable connectivity among areas. Fossil data hold considerable information about past distribution of lineages, but suffer from largely incomplete sampling. Here we present a new dispersal–extinction–sampling (DES) model, which estimates biogeographic parameters using fossil occurrences instead of phylogenetic trees. The model estimates dispersal and extinction rates while explicitly accounting for the incompleteness of the fossil record. Rates can vary between areas and through time, thus providing the opportunity to assess complex scenarios of biogeographic evolution. We implement the DES model in a Bayesian framework and demonstrate through simulations that it can accurately infer all the relevant parameters. We demonstrate the use of our model by analysing the Cenozoic fossil record of land plants and inferring dispersal and extinction rates across Eurasia and North America. Our results show that biogeographic range evolution is not a time-homogeneous process, as assumed in most phylogenetic analyses, but varies through time and between areas. In our empirical assessment, this is shown by the striking predominance of plant dispersals from Eurasia into North America during the Eocene climatic cooling, followed by a shift in the opposite direction, and finally, a balance in biotic interchange since the middle Miocene. We conclude by discussing the potential of fossil-based analyses to test biogeographic hypotheses and improve phylogenetic methods in historical biogeography.

## 1. Introduction

Global biodiversity has undergone numerous changes of different magnitude since the origin of life [1–3] and these variations ultimately result from the interplay between two processes: speciation and extinction [4,5]. At smaller scale, when considering a delimited geographical area, such as a continent, island or mountain range, diversity dynamics are further governed by the geographical range evolution or organisms. More specifically, the biota of a given area is not only influenced by speciation and extinction processes, but also by immigration of species from adjacent or distant areas and by local extinction events, driven, for example, by the complete emigration of a lineage [6–9]. Thus, understanding biogeographic history and the biotic connectivity among areas is crucial to explain how present spatial patterns of diversity were shaped [10–12] and to test whether past migration dynamics of organisms conform to the expectations derived from geological and climatic models [13,14].

Tracing the biogeographic history of taxa is, however, a challenging task because, while we can be reasonably confident in the present distribution of many organisms, it is difficult to infer where extant and extinct taxa occurred in the past [15,16]. Over the last two decades, several methods have been developed to make inferences in historical biogeography, using phylogenetic data and explicit models of geographical range evolution [17–22]. Among these, parametric methods transformed biogeographic reconstructions, traditionally based on cladistic assumptions, into more rigorous model-based probabilistic inferences [15].

Most modern methods implement likelihood-based approaches on dated phylogenies of extant taxa to infer historical biogeography with a focus on the estimation of ancestral geographical ranges at the nodes of a tree. The first, and perhaps most widely used, likelihood-based approach in historical biogeography is the dispersal–extinction–cladogenesis model (DEC; [18,20]). Under this model, geographical ranges change across a phylogenetic tree by cladogenetic events and anagenetic range evolution. Cladogenetic events describe the inheritance of an ancestral range by two descendent lineages, based on where the speciation event occurs, i.e. within an area or between areas [18,23]. Anagenetic range evolution includes all the events that alter the distribution of a lineage through time, either by range expansion through dispersals (*sensu* [17]) or by range contraction, through local extinction or extirpation [23]. Dispersal and local extinction events are modelled as the result of a continuous time Markov process with rate parameters estimated from the data [20].

Since the introduction of the DEC approach, models of range evolution have become richer in number of parameters, allowing users to test the relative importance of different speciation modes such as sympatric speciation, vicariance and founder-event speciation [22]. These additional parameters provide a comprehensive framework to statistically assess the most likely scenarios of range inheritance at cladogenetic events. By contrast, anagenetic range evolution is still typically modelled under very simplistic parametrizations involving two parameters: one rate of dispersal (or area gain) and one rate of local extinction (or area loss) [20–22]. Most phylogenetic methods are, therefore, currently unable to infer rate asymmetries and temporal variations in dispersal and extinction from the data (but see [10,24,25]). This limitation can be attributed to the fact that, although it is theoretically possible to populate the DEC transition matrix with asymmetric dispersal rates and area-specific extinction rates [20], the data used in biogeographic analysis (current ranges and phylogenetic relationships of extant species) are probably insufficient to estimate all required parameters [26]. This is highlighted by the poor accuracy of dispersal and extinction rates estimated under DEC-based methods, even in the simple case of constant and symmetric parameters [20,22].

Improved estimation of dispersal and extinction rates is potentially achieved by integrating the processes of geographical range evolution within the birth–death diversification process as in the GeoSSE model, which allows the estimation of area-specific dispersal, extinction and speciation rates [27–29]. However, obtaining accurate and reliable parameter estimates under this model can be problematic [30] and requires large phylogenies involving hundreds of taxa [27,31], thus restricting the applicability of GeoSSE to clades that are today very diverse and well sampled. Complex models of anagenetic range evolution can also be inferred after combining several phylogenetic datasets and assuming that they evolve under similar biogeographic settings [10,11,13]. For instance, the joint analysis of multiple clades can be used to infer overall biotic connectivity among areas (quantified by dispersal rates) and their carrying capacities in a Bayesian framework [11]. Even these complex models, however, typically make the unrealistic assumption that dispersal and extinction processes are time-homogeneous with constant rates through time.

Recent studies have shown that the inclusion of fossil information in phylogeny-based biogeographic analyses can significantly improve the estimation of ancestral ranges and their evolution [16,23,29,32]. These methods, however, often rely on a known phylogenetic placement of extinct taxa, which can be reconstructed only when fossil morphology is sufficiently well preserved and phylogenetically informative. Unfortunately, this is seldom the case for the majority of taxa. Furthermore, the integration of fossils in phylogeny-based biogeographic analyses does not explicitly model the process of fossil preservation, thus neglecting its inherent sampling biases.

To tackle the methodological limitations outlined above, we develop here a new probabilistic model, which we term the ‘dispersal–extinction–sampling’ model (DES). This model estimates the parameters of anagenetic geographical evolution (dispersal and extinction) using exclusively fossil occurrence data and without using phylogenetic information. Compared with most phylogenetic methods, our approach implements a more realistic model of range evolution, in which rates of dispersal and extinction can vary across areas and through time. Furthermore, we introduce an explicit model of preservation in order to account for the sampling biases inevitably linked to the incompleteness of the fossil record. The lack of an underlying phylogeny makes the DES model unsuitable for ancestral range estimation, but applicable to a wide range of extinct and extant lineages for which fossil occurrences are available, including those lacking a reliable phylogenetic hypothesis. The main focus of the DES model is the estimation of dispersal rates between areas and area-specific extinction rates, with the possibility to allow for temporal rate variations.

In this study, we (i) present the DES model and provide a Bayesian implementation to infer dispersal, extinction and sampling parameters, (ii) assess its ability to accurately estimate the parameters through extensive simulations and (iii) apply the method to a large empirical dataset of plant fossils to estimate dispersal and extinction levels in North America and Eurasia throughout the Cenozoic. Finally, we discuss the usefulness of dispersal and extinction rates estimated from fossil data to inform and improve phylogeny-based biogeographic inferences.

## 2. Material and methods

We consider a system of discrete areas and use stochastic processes of dispersal, extinction and sampling to model geographical range evolution of extinct and extant lineages. Our model of biogeographic evolution is largely based on the formulation and terminology first developed in a phylogenetic context within the DEC framework [18,20]. Thus, as in the DEC model, dispersal events indicate episodes of range expansion, while extinction stands for local extirpation, which yields range contraction. In our notation, extirpation from all areas leads to empty geographical range and corresponds to the complete extinction of a lineage. The DES model is implemented in Python [33] and is available as part of the open-source package PyRate [34]: https://github.com/dsilvestro/PyRate.

### (a) Coding fossil geographical ranges

The model described here is restricted to two discrete areas indicated by *A* and *B*, but it could be extended to multiple areas in future implementations. The geographical range of a taxon is coded by its presence or absence within areas. The possible geographical ranges in a system of two areas are 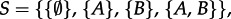 where 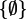 indicates that a lineage is absent from both areas and {*A*, *B*} indicates that a lineage is present in both areas.

**Figure 1.**
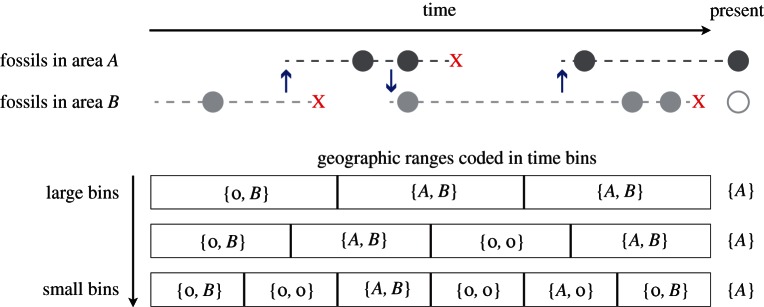
Effect of different time bins on the coding of biogeographic ranges through time. Dashed lines indicate the true geographical history of the lineage, involving three dispersals (arrows) and three extinction events (crosses). Circles indicate the sampled fossil occurrences, the empty circle at the present indicates that the taxon is currently absent from area *B*. The sampled ancestral states (indicated with *O* in equations (2.4), (2.5)) are here coded using large, intermediate and small time bins and shown at the bottom part of the plot.

Let us consider a taxon *i* (e.g. a species or genus) for which fossil occurrences of different ages were found in areas *A* and *B* (figure 1). We score its observed geographical range based on the distribution of sampled fossils within discrete time bins of equal size. In cases of exceptional preservation, such as with some marine planktonic microrganisms or pollen records, it is possible to assess the presence or absence of a taxon almost continuously through time, by examining its fossil records [35,36]. In most cases, however, fossil occurrences represent instantaneous information about the presence of a taxon in a locality and are separated by time intervals during which no records are available, potentially due to incomplete sampling. As the absence of a lineage from the fossil record of an area may be the result of incomplete sampling (and not necessarily a true absence), we indicate such putative absences with 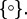 Thus, if a taxon *i* was sampled at time bin *t* only in area *A*, its observed geographical range is indicated by 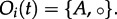 We consider the geographical ranges of extant taxa (both the presence and absence) to be known. Given a set of fossil occurrences, the biogeographic ranges of a taxon can be coded differently depending on the size of the time bins, as shown in figure 1. Decreasing bin size increases the frequency of time slices with empty ranges (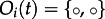) and decreases the number of time slices with widespread ranges (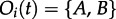; figure 1). We explore the effects of different bin sizes on the estimated biogeographic parameters through simulations (see below).

### (b) Probabilistic model

We model the process of geographical range evolution as a stochastic Markov process, in which the range of a taxon can expand to a new area through dispersal events and contract by local extinction events determining the disappearance of the taxon from an area. This model of biogeographic evolution corresponds to the anagenetic component of the DEC model [18,20]. As in the DEC implementation, we construct a transition matrix *Q* based on the dispersal and local extinction rates, and use it to calculate the probability of range transition as a function of a time interval Δ*t* [20]:
2.1
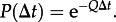
The *Q* matrix for a system of two areas involves four rate parameters:
2.2
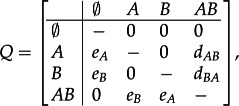
where *d_AB_* is the rate of dispersal from area *A* to area *B*, *d_BA_* is the rate of dispersal from area *B* to area *A*, and *e_A_*, *e_B_* are the local extinction rates in areas *A* and *B*, respectively. As the rates from 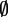 to any other area are set to 0, lineages are considered to have gone globally extinct when their range is empty. Dispersal and extinction rates quantify the expected number of dispersal and extinction events per lineage per time unit (e.g. millions of years (Myr)), respectively.

The incompleteness of the fossil record can bias the observed range evolution. That is, the absence of a lineage from an area at time *t* may mean that (i) the organism did not occur in that area at the time (true absence) or (ii) the organism occurred in the area but did not produce any known fossil records (pseudo-absence). Thus, the observed range 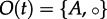 may correspond to two true ranges: *R*(*t*) = {*A*} or *R*(*t*) = {*A*, *B*}. While sampling biases affect our interpretation of absences, we assume the presence of a lineage in an area in the observed range to be true. For instance, a lineage with observed range 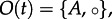 is assumed to be present in area *A*, while it may or may not be present in *B*.

Estimating dispersal and extinction rates from fossil data require that we account for their incompleteness in the model, as in the case of other macroevolutionary parameters [37–39]. We define the preservation process as a set of multiple processes including the fossilization of an organism, its modern day sampling and taxonomic identification. Under a Poisson process of preservation, the preservation rate *q* quantifies the expected number of fossil occurrences per lineage per Myr [39]. To incorporate sampling biases in the inference of dispersal and extinction rates, we introduce two parameters (*q_A_*, *q_B_*) expressing area-specific preservation rates. Given a homogeneous Poisson process of fossil preservation with rate *q_A_* (or *q_B_*), we can calculate the probability that a lineage present in area *A* (or *B*) did not leave any fossil records within a time bin. This scenario represents a false absence in that the presence of a lineage in an area is not observed as the result of fossil incompleteness. The probability of a false absence in area *A* in a time bin of size Δ*t* is:
2.3
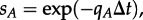
where exp(−*q*Δ*t*) is the probability of a waiting time (Δ*t*) without fossil occurrences. A similar equation applies to area *B* based on the preservation rate *q_B_*. By contrast, the probability of a true absence, whereby a lineage that is not observed in area *A* (or *B*) indeed did not occur in the area, is simply given by 1 − *s_A_* (or 1 − *s_B_*). In our implementation the preservation rates (*q_A_*, *q_B_*) are treated as unknown variables and estimated from the data. The probabilities of false and true absences in a given area are therefore a function of both preservation rates (*q*) and bin size (Δ*t*).

The likelihood of a geographical range *R*(*t*_1_) conditional on a range *R*(*t*_0_) is
2.4
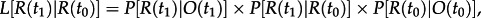
where 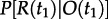 and 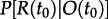 are the probabilities of a range given the observed ranges calculated as shown in table 1, and 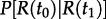 is the probability of range transition calculated by exponentiation of the *Q* matrix (equations (2.1), (2.2)) times the elapsed time 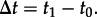 This formulation of the likelihood is equivalent to that of the DEC model (equation (2.1) in [18]), except that the probabilities of the ancestral ranges are not set to be uniform, but are based on the ranges observed in the fossil record and on the preservation rates (table 1). As in the DEC framework, we consider the geographical ranges at the present to be known without sampling biases, so that the probability of a range at the present (*R*(*t_p_*)) is 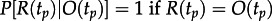 and 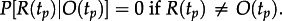 Note that for extinct lineages, the geographical range is set to 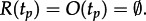 For a given vector of observed geographical ranges for a lineage 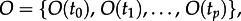 where *t*_0_ represents the time of first appearance of the lineage, we calculate the likelihood 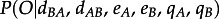 recursively from *t_p_* to *t*_0_ while considering all possible ancestral states at times 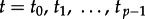 and their probabilities [18,20]. We consider geographical ranges to evolve independently across different lineages, thus the likelihood of a dataset of *N* lineages (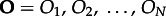) is calculated as the product of the likelihoods of all lineages:
2.5
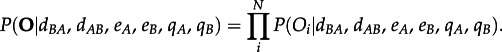

**Table 1.** Observed ranges, true ranges and their probabilities based on the preservation process. Based on a preservation rate *q*, we can quantify the probability of false absences deriving from the incompleteness of the fossil record (*s_A_*, *s_B_*; equation (2.3)) and the probability of true absences (1 − *s_A_*, 1−*s_B_*).

observed range (*O*)	true range (*R*)	probability (*P*[*R*|*O*])
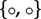	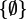	(1 − *s_A_*) (1 − *s_B_*)
	{*A*}	*s_A_*(1 − *s_B_*)
	{*B*}	(1 − *s_A_*)*s_B_*
	{*A*, *B*}	*s_A_s_B_*
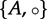	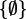	0
	{*A*}	(1 − *s_B_*)
	{*B*}	0
	{*A*, *B*}	*s_B_*
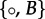	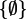	0
	{*A*}	0
	{*B*}	(1 − *s_A_*)
	{*A*, *B*}	*s_A_*
{*A*, *B*}	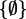	0
	{*A*}	0
	{*B*}	0
	{*A*, *B*}	1

We consider taxa to be independent despite the fact that they may be phylogenetically connected with other lineages in the dataset (as in [40]). However, we emphasize that the recursive likelihood calculation starts at the present (*t*_p_) and stops at the first appearance of a lineage (its oldest fossil, *t*_0_) without attempting to infer its true origination time and initial ancestral range. While the range of a lineage at its origin (which most probably precedes the oldest fossil) is not independent of the distribution of its ancestor, the anagenetic geographical evolution following the first appearance can be considered as an independent instance of geographical evolution [18,40]. Additionally, the sparsity of the fossil record for most taxonomic groups implies that only a fraction of all lineages are preserved and sampled, thus making even the initial geographical range of the sampled lineages essentially independent of all others.

### (c) Bayesian implementation

We implemented the DES model in a Bayesian framework and used a Markov Chain Monte Carlo (MCMC) algorithm to sample the dispersal, extinction and sampling parameters from their posterior distribution. We used an exponential prior on the rates 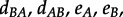 with rate parameter *g*. Because the dispersal and extinction rates can take any positive value, the definition of appropriate priors might be subjective. To overcome this difficulty, we considered the rate *g* as an unknown variable with a gamma hyper-prior 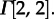 This allowed us to estimate the parameter of the prior distribution Exp(*g*) directly from the data (see below). We used a beta distribution *B*[1,1] as flat prior on the probabilities of false presence, thus assigning equal prior probabilities to any 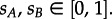 This effectively corresponds to an exponential prior distribution on the preservation rates *q_A_*, *q_B_* with rate *λ* = 1/Δ*t*. The posterior probability of the DES parameters given a dataset of fossil occurrences **O** is
2.6
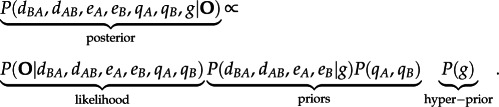


We used multiplier proposals for dispersal and extinction rates and uniform proposals with reflection at the boundaries 0 and 1 [41] for the probabilities *s_A_*, *s_B_* from which we derived the preservation rates. We implemented posterior sampling using the standard Metropolis–Hastings algorithm [42,43] based on the posterior ratio and the proposal ratio. The hyper-parameter *g* was sampled directly from the conjugate posterior distribution:
2.7
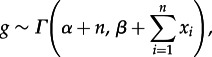
where *n* is the number of dispersal and extinction parameters (*n* = 4 in a model with two geographical areas) and *x* is the vector of dispersal and extinction rates.

### (d) Simulations and assessment of accuracy and precision

We assessed the performance of the DES model through simulations and quantified how close the rate estimates are to the respective true values (accuracy) and the degree of uncertainty around the estimates (precision). In order to explore different properties of the model, we used a wide range of settings by varying dispersal, extinction and preservation rates, and also the size of the dataset and the size of the time bins used to code the geographical ranges. The simulation process required three steps. We first simulated the anagenetic evolution of geographical ranges along a lineage based on a continuous time Markov process with rates *d_BA_*, *d_AB_*, *e_A_*, *e_B_*, and on a random initial range. Second, based on the amount of time spent by the lineage in each area and on the preservation rates (*q_A_*, *q_B_*), we simulated fossil occurrences using a homogeneous Poisson process. Finally, we coded the observed ancestral geographical ranges within time bins as shown in figure 1. We repeated this procedure to generate datasets with multiple lineages. In each simulation, we drew the model parameters from uniform distributions: 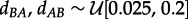 (dispersal rates), 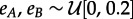 (extinction rates), 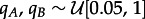 (preservation rates), 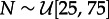 (number of lineages). We determined the probabilities of sampling a fossil occurrence within each time bin 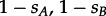 based on the preservation rates *q_A_*, *q_B_*, and on the length of the time bins, using equation (2.3). For instance, with a preservation rate *q* = 0.1 (i.e. one expected fossil occurrence per lineage every 10 Myr), the resulting sampling probabilities for bins of size 5, 2.5, 1 and 0.5 Myr are 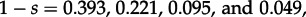 respectively. All simulated datasets spanned 50 Myr. In the implementation of the DES model described above, only the anagenetic geographical history following the oldest fossil occurrence of a taxon is considered, whereas its true time of origin and the processes of origination and range inheritance at speciation are ignored. As the DES model treats each lineage as an independent instance of anagenetic evolution and does not make any assumptions about an underlying phylogenetic structure linking taxa, origination times do not need to follow a birth process. Thus, we drew the origination times of lineages from a uniform distribution 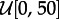 and generated 1200 simulations with the same random settings equally split into four groups defined by the size of the time bins used as units for the likelihood calculation. The size of the time bins were set to 5, 2.5, 1 and 0.5 Myr (yielding 10, 20, 50 and 100 bins, respectively).

We analysed the simulated datasets by running 26 000 MCMC iterations, sampling every 10 iterations. After assessing the convergence of the MCMCs using Tracer v. 1.6 [44] and excluding the first 6000 iterations as burn-in, we summarized the posterior samples of all parameters by calculating the modal value from their frequency distribution as a proxy for maximum *a posteriori* (MAP) and 95% highest posterior density (HPD). We preferred the MAP value over the mean because the posterior distributions of the rates are strongly skewed due to the hard boundary at 0 [45]. The overall accuracy of the estimated rates across simulations was quantified as mean absolute percentage error (MAPE) calculated as
2.8
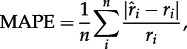
where *n* is the number of simulations, *r_i_* is the parameter value used to simulate the dataset and 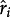 is the posterior estimate of the parameter. We also checked whether the rate asymmetries were accurately inferred by assessing the differences between dispersal rates in the two directions (from *A* to *B* and from *B* to *A*) and between extinction and preservation rates in the two areas. Rate asymmetries were expressed as the logarithm of the ratios between rates, e.g. 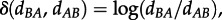 thus 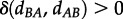 when 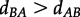 and 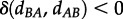 when 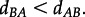 We, therefore, plotted the estimated log ratios 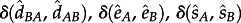 against the true log ratios 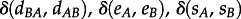 to visually detect biases in our posterior estimates of asymmetric rates. To further explore the existence of biases in the parameter estimates, we calculated the percentage errors (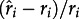) for dispersal and extinction rates and plotted them against the number of lineages in the dataset (*N*), the minimum preservation rate (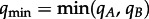) and asymmetry of the preservation rates 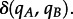


The precision of the posterior rate estimates was assessed by the size of the 95% HPD relative to the parameter value:
2.9
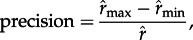
where 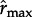 and 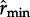 define the boundaries of the 95% HPD range for a given parameter *r*. We plotted the relative HPD size of dispersal as an extinction rate against *N* and *q*_min_ to determine whether the precision of these parameters is sensitive to the size of the dataset and to preservation rates.

### (e) Temporal rate variation

The DES model described above assumes a uniform process of dispersal and extinction and constant preservation rates through time. As this assumption is probably not realistic (given, for example, the association of climate with fossilization rates), we relaxed it by introducing a ‘stratified DES model’ (cf. [18]), which allows us to define time frames characterized by independent sets of DES parameters. Each time frame *T* is delimited by fixed user-defined times of rate shift and is assigned its own *Q_T_* matrix (with independent 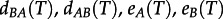 rates) and specific preservation rates (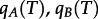). Thus, the stratified DES model involves the estimation of additional dispersal, extinction and preservation rates from the data. This is obtained by introducing different *Q* matrices and preservation rates in the recursive calculation of the likelihood described above as a function of time. We assumed a single exponential prior distribution on the dispersal and extinction rates with hyper-parameter *g* shared across all time frames, in order to reduce the risk of over parametrization [46].

The stratified DES model differs from the standard implementation of stratified DEC models, which is based instead on *a priori*-defined constraints that change the probability of dispersal between areas through time. Such dispersal constraints in DEC are user-defined and applied as multipliers to a single estimated dispersal parameter and, therefore, do not involve the addition of free parameters [20,22].

In order to compare the fit of stratified and constant rate models, we implemented the thermodynamic integration algorithm [47] to compute the marginal likelihood of each model. We sampled likelihood values along a path ranging from the posterior to the prior by altering the MCMC acceptance probability by a factor 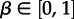 [47]. We used 10 *β* values obtained from a beta distribution *B*(0.3,1) [48,49] and integrated the mean of the sampled likelihoods to obtained marginal log-likelihood of a model. We calculated log Bayes factors as the difference between marginal log-likelihoods [50].

### (f) Cenozoic vascular plants

We tested the stratified DES model on a large dataset of vascular plants based on the data recently compiled by Silvestro *et al.* [46]. The data were originally obtained from the Paleobiology Database (http://paleobiodb.org) using multiple search queries in order to obtain the most comprehensive dataset of the plant fossils and only included occurrences identified to the species or genus level [46]. We considered all available Cenozoic fossils of non-marine vascular plants sampled in North America and Eurasia, which represent the large majority of all occurrences [12], resulting in a final dataset of 10 276 occurrences and 273 genera (99 of which are extinct). The incomplete nature of fossilized plant organs poses strong difficulties in confidently assessing the taxonomic placement of fossil occurrences at species level [46,51] and more than 2000 records in our dataset are only identified at genus level. Furthermore, fossil incompleteness and difficult species identification may explain to a large extent why only 22 of the 1452 species in our dataset were sampled in both North America and Eurasia. For these reasons, we used genera as the taxonomic units in our analysis. The genera along with the number of occurrences recorded in each area are listed in the electronic supplementary material, tables S1–S7. We used North America and Eurasia (figure 6) as two discrete areas for the DES analysis and coded the fossil geographical ranges using time bins of 2.5 Myr, a length that appeared adequate based on our simulations and the degree of uncertainty around the age estimates of the fossil occurrences (see below). Fossils were evenly distributed between the two areas with 5274 and 5002 occurrences, respectively (electronic supplementary material, figures S7 and S8). We assigned the fossil occurrences to the continents based on the country in which they were collected and assigned present day genera ranges based on the world checklist of selected plant families [52] complemented with regional and national floras [53–59] when necessary. We excluded occurrences solely based on human introduction and used the Global Biodiversity Information Facility [60] to assign the ranges for a few genera where no information could be obtained from the floras. The final dataset is available at https://github.com/dsilvestro/PyRate or upon request.

Most fossil occurrences (99.8% in our dataset) were not provided with a single age estimate, but included temporal ranges (minimum and maximum ages). Such ranges typically derive from the upper and lower temporal boundaries of the stratigraphic units to which fossils were assigned. The average temporal range measured 7.68 Myr and 95% of them spanned between 1 and 22 Myr. We treated these temporal ranges as uniform distributions describing the uncertainty around the age estimated for each fossil occurrence [46]. Thus, rather than just using the mid point age in our analyses, we randomly resampled the ages of each fossil occurrence from the respective temporal range.

We ran two initial analyses using the mean age of each fossil occurrence. In the first analysis, we assumed a constant rate model, while in the second we tested the stratified model after defining four time frames based on global climatic trends [61]: 66–50 Ma (warming), 50–32 Ma (cooling), 32–14 Ma (stable), 14–0 Ma (cooling). We acknowledge that these periods contained several smaller, though potentially significant, climatic fluctuations (e.g. the Paleocene–Eocene Thermal Maximum at *ca* 56 Ma). However, increasing the number of time frames would increase analytical complexity and, more importantly, require a higher temporal precision and abundance of the fossil data than currently available.

We ran 100 000 MCMC iterations sampling for every 100, to obtain posterior samples of the DES parameters. In order to compare the fit of the two models, we calculated their marginal likelihood by running 500 000 MCMC iterations under the thermodynamic integration algorithm described above.

To incorporate the dating uncertainties, we randomly resampled the ages of each fossil occurrence (as described above) 100 times, each time re-coding the observed fossil geographical ranges (**O**), and repeated the DES analysis under the stratified model on all replicates. The joint results of all analyses yielded the final posterior intervals of the dispersal, extinction and preservation rates. Dispersal and extinction rates were considered significantly asymmetric between areas when 0 fell outside the 95% HPD of the difference between their posterior samples.

## 3. Results

### (a) Parameter estimation

**Figure 2.**
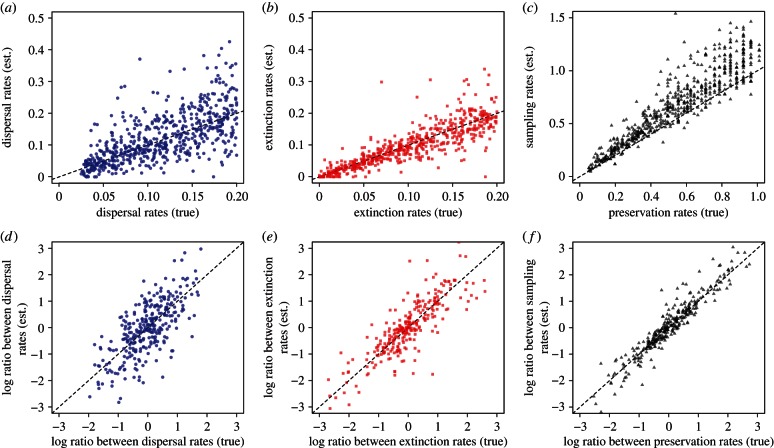
Dispersal, extinction and preservation rates obtained from simulations, using time bins of 2.5 Myr (*a*–*c*). True rates (used to simulate the data) are plotted against estimated rates (maximum *a posteriori*). Points below the diagonal (dashed line) represent underestimates, points above the diagonal represent overestimates. The ability of the model to recover rate asymmetry is shown by plotting the log ratio between the true rates against the log ratio between estimated rates (*d*–*f*).

The parameters of the DES model, i.e. dispersal, extinction and preservation rates, are accurately estimated in our framework, with MAPE below 0.5 under most simulation settings (table 2). Simulations generated under different bin sizes to code fossil geographical ranges (cf. figure 1) revealed that large bin size (5 Myr) leads to low accuracy of the preservation rates, which are otherwise accurately inferred under smaller bins. Bin size only has a moderate effect on the accuracy of dispersal and extinction rate estimates, with effects generally smaller than 0.1 in terms of MAPE (table 2; figure 2*a*–*c*; electronic supplementary material, figures S1–S3). The parameters were estimated with similarly high accuracy using bins of size 2.5, 1 and 0.5 Myr, with an overall MAPE < 0.4.

**Table 2.** Accuracy of the rate estimates under the DES model, using different bin sizes for coding the geographical ranges. Mean absolute percentage errors (MAPE) of estimated dispersal, extinction and preservation rates were calculated over 300 simulations for each bin size (standard deviations are given in parentheses). MAPE ranged between 0.2 and 0.5 depending on the parameter (with the exception of the preservation parameters in datasets with bin size = 5), but were quite consistent across different bin sizes. The smallest MAPE, overall, was obtained under time bins of 2.5 Myr.

bins	bin size	all parameters	dispersal	extinction	preservation
10	5	0.668 (0.368)	0.496 (0.816)	0.418 (0.813)	1.09 (1.099)
20	2.5	0.363 (0.06)	0.427 (0.468)	0.309 (0.564)	0.353 (0.482)
50	1	0.345 (0.099)	0.412 (0.719)	0.393 (0.941)	0.231 (0.282)
100	0.5	0.348 (0.094)	0.424 (0.456)	0.377 (0.627)	0.242 (0.337)

**Figure 3.**
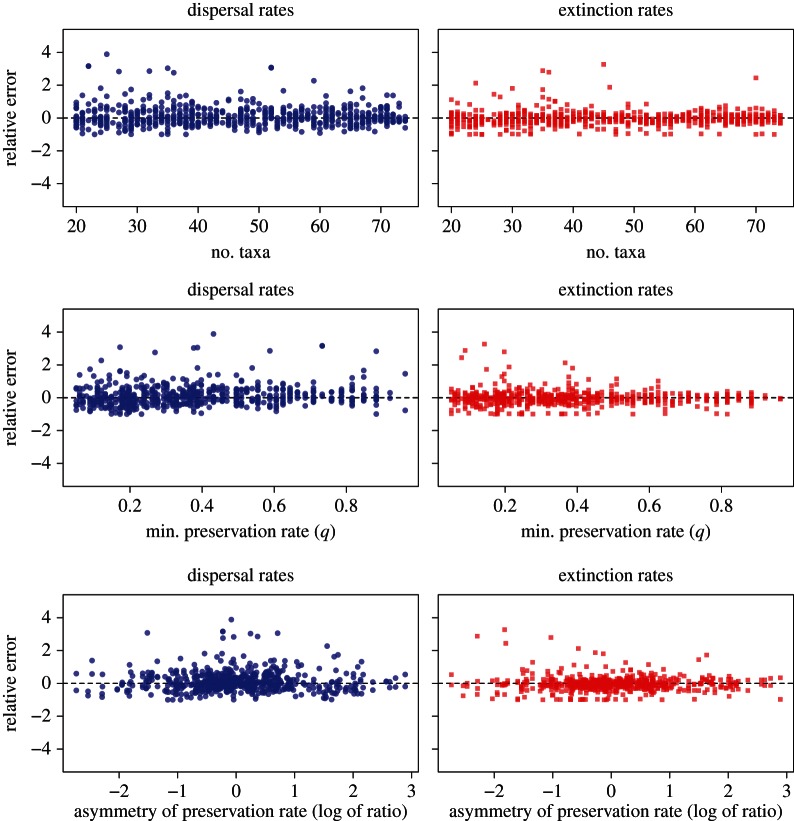
Relative errors of the estimated dispersal and extinction rates (maximum *a posteriori*) obtained from simulations, using time bins of 2.5 Myr. Relative errors are calculated against the true rates (used to simulate the data). Thus, values close to 0 indicate accurate estimates, whereas positive and negative values indicate overestimation and underestimation of the rates, respectively.

Rate asymmetries are correctly inferred, despite some stochastic errors (more pronounced for dispersal rates; figure 2*d*–*f*; electronic supplementary material, figures S1–S3). The estimated dispersal and extinction rates do not show any consistent biases towards overestimation or underestimation and percentage errors show that the accuracy of the estimates is mostly unaffected by the number of taxa, minimum preservation rates and asymmetries of preservation (figure 3). The preservation rates, despite being estimated with high accuracy (with bin size less than 5 Myr), tend to be slightly overestimated (figure 2*c*; electronic supplementary material, figures S1–S3).

**Figure 4.**
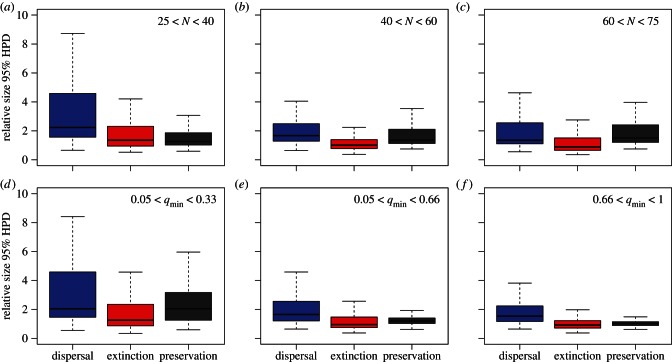
Relative size of the 95% credible intervals (HPD) around the estimated dispersal, extinction and preservation rates. Estimates are based on time bins of 2.5 Myr to code the fossil geographical ranges. The relative sizes of the HPDs are summarized over 300 simulations and split by the size of the datasets (*N* number of lineages; *a*–*c*) and by the minimum preservation rate (*q*_min_; *d*–*f*).

The relative size of the HPDs reveals that the amount of uncertainty around the parameter estimates is generally of the same order of magnitude as the parameter value (figure 4). The largest relative HPD ranges are found for dispersal or for preservation rates, depending on the settings. The precision of the rate estimates improves substantially with increasing size of the datasets (e.g. more than 40 lineages; figure 4*a*–*c*) and with higher preservation rates (e.g. *q*_min_ > 0.33; figure 4*d*–*f*). The bin sizes used to code the fossil geographical ranges may have a strong impact on the precision of the parameter estimates. Large bins (5 Myr) resulted in low precision around dispersal and extinction estimates (electronic supplementary material, figure S3). When using small bins (1 or 0.5 Myr) dispersal and extinction rates are more precise, but larger uncertainties are inferred around the preservation rates (electronic supplementary material, figures S4 and S5). Considering both accuracy and precision, the analyses indicate that the DES model performs best with time bins of 2.5 Myr under our simulation settings. Thus, we used 2.5 Myr time bins in the subsequent analysis of empirical data.

### (b) Cenozoic dispersals and extinctions in vascular plants

The time-stratified DES model (marginal log-likelihood = −4803.63) fits the data significantly better than the constant rate model (marginal log-likelihood = −4954.83). The resulting Bayes factor equals 151.2 log units in favour of the stratified model, which can be interpreted as very strong statistical support [50]. The parameter estimates under the stratified model fell in the range of values used in our simulations, but the credible intervals were narrower than in most simulations, probably as a consequence of the large size of the empirical dataset. The preservation rates ranged between 0.28 and 0.75 in North America, depending on the time period, and between 0.29 and 0.66 in Eurasia. These values correspond to preservation rates (*q*) ranging from 0.13 to 0.56 expected occurrences per lineage per Myr, respectively.

**Figure 5.**
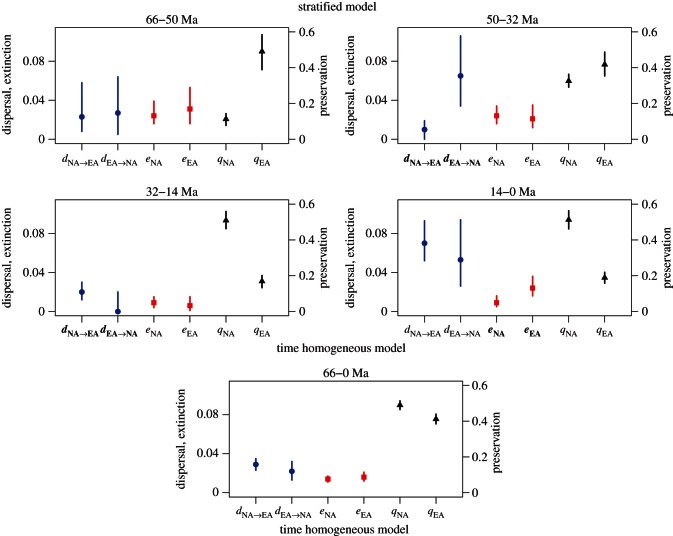
Results of the DES analyses on the Cenozoic fossil record of vascular plants in North America (NA) and Eurasia (EA) for the stratified and time-homogeneous models. Posterior estimates (MAP) of the dispersal (blue), extinction (red) and preservation (black) rates are obtained after combining 100 replicates to account for dating uncertainties in the fossil record with whiskers indicating the 95% credible intervals. Dispersal and extinction rates (left *y*-axis labels) correspond to the expected number of events per lineage per Myr, preservation rates (right *y*-axis labels) express the expected number of fossil occurrences per lineage per Myr. Dispersal and extinction rates highlighted in bold (*x*-axis labels) indicate significant asymmetries between areas.

**Figure 6.**
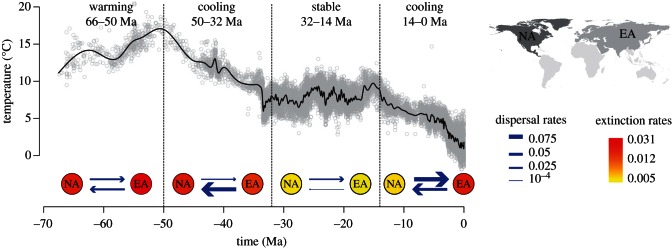
Cenozoic dispersal and extinction rates of vascular plants in North America (NA) and Eurasia (EA). Posterior estimates of the dispersal and extinction rates are calculated within four time frames, after combining 100 replicates to account for dating uncertainties in the fossil record. The temperature curve was obtained from Zachos *et al.* [61].

Dispersal rates were roughly symmetric during the early Cenozoic (66–50 Ma), but underwent substantial changes between 50 and 32 Ma resulting in strongly asymmetric rates (figures 5 and 6). The dispersal rate from North America to Eurasia drastically decreased to less than half, whereas dispersal rate in the opposite direction underwent a 2.4-fold increase. Frequency of dispersal from North America to Eurasia increased again between 32 and 14 Ma, while it dropped to almost zero from Eurasia to North America. Finally, dispersal rates in both directions substantially increased towards the recent (14–0 Ma).

Extinction rates (expected number of extinction events per lineage/Myr) were similar in the two continents and ranged between 0.02 and 0.03 from the early Cenozoic to 32 Ma (figures 5 and 6). Subsequently, they dropped by 1 order of magnitude similarly in both areas between 32 and 14 Ma. The low extinction rate remained approximately unchanged until the present in North America. By contrast, we inferred a fourfold increase in extinction rate in Eurasia between 14 Ma and the present, resulting in significantly different extinction rates between the two areas in this time period.

## 4. Discussion

### (a) Properties and assumptions of the dispersal–extinction–sampling model

We developed a new approach to infer the dynamics of geographical range evolution from fossil data, by adapting and expanding the dispersal and extinction stochastic process previously described in a phylogenetic framework [18]. Rate estimates under the DES model were accurate across the scenarios tested through simulations. These included a wide range of fossil preservation rates, under which the expected number of fossil occurrences per lineage varied between 1 every Myr (*q* = 1) and 1 every 20 Myr (*q* = 0.05). These preservation rates are realistic for several taxonomic groups, as shown by previous empirical analyses [46,62].

The use of discrete time bins in our implementation facilitates the coding of the observed geographical range of fossil taxa and the estimation of the sampling parameters. We highlight, however, that the underlying dispersal and extinction processes are modelled as (and were simulated under) continuous time Markov processes. While the definition of time bins to code geographical ranges represents a somewhat arbitrary step in setting up a DES analysis, we showed that the use of different binning has limited effects on parameter estimation. Large bins (5 Myr) yielded slightly lower accuracy of dispersal and extinction rates (table 2). This is due to the fact that many true dispersal and extinction events are likely to become unobserved when geographical ranges are coded with large bins, as this procedure will lead to frequent widespread distributions (figure 1). This is also a likely reason for the low accuracy in the preservation rates under large bins, which is mainly linked with their overestimation. In addition, analyses carried out under large bins yielded larger credible intervals in the case of small datasets and low preservation rates (electronic supplementary material, figure S5), suggesting substantial levels of uncertainty around the dispersal and extinction parameters. By contrast, the use of smaller bins (between 2.5 and 0.5 Myr) yielded higher levels of accuracy indicating that the choice of bin size within this range has little effect on the reliability of the results, though the precision of the preservation rate decreased with smaller bins.

As a general rule, the selection of bin size should reflect the density of fossil records, their dating accuracy and the time-scale of interest. For instance, a dataset of densely sampled and accurately dated fossil occurrences spanning few millions of years might allow for time bins of 10 000 years or similar order of magnitude. The use of small equal-size time bins, instead of stratigraphic time units, provides us with the opportunity to incorporate dating uncertainties in the estimation of dispersal and extinction rates. As we showed with the analysis of the plant fossil record, the ages of fossil occurrences can be resampled within the respective stratigraphic ranges. This resampling procedure will only have an effect on the pattern of observed ancestral ranges of a lineage (*O_i_*) if the size of the time bins is sufficiently small compared with temporal ranges attributed to fossil occurrences.

Our simulations indicated that extinction rates are estimated with higher accuracy and precision than dispersal rates (e.g. figures 2 and 4), contrary to what has been observed in phylogeny-based analyses under DEC [20], where extinction is most severely biased. A potential reason for this pattern is that, under the settings used in our simulations, the number of extinction events might exceed the number of dispersal events. Indeed, while both rates were sampled from the same distribution (see Material and methods), several lineages may go extinct before they have the chance of dispersing to a new area. Additionally, fossil data provide a more direct evidence of extinction, for instance, in cases of lineages with fossil occurrences in areas where the lineage does not occur at the present (e.g. area B in figure 1).

In the DES model, we use the preservation rates to estimate the probability of false absences due to the incompleteness of the fossil record, and these parameters are essential to correctly estimating dispersal and extinction rates. Preservation rates can only be inferred from lineages that were sampled in at least one fossil occurrence, thus neglecting lineages that are entirely missing from the fossil record [39]. This fact might explain the slight tendency toward an overestimation of the preservation rates (figure 2*c*).

While the DES model allows for asymmetric dispersal and extinction rates, it assumes that (i) the same rates apply to all lineages and (ii) the chance of dispersal and extinction of a lineage exclusively depends on its geographical distribution coded as the presence or absence in discrete areas. The two assumptions could be relaxed in future developments of the model. The first issue can be addressed by introducing rate heterogeneity across lineages, for instance, based on the Gamma models already implemented for nucleotide substitutions [63] and fossil preservation [39]. The second assumption is linked to coding geographical ranges as the presence or absence within discrete areas and its inherent limitations. This approach inevitably ignores important factors such as the abundance of a taxon (e.g. population size) and its distribution within the discrete areas, which almost certainly affect the biogeographic history of a lineage. In case of very well-preserved fossil lineages with many occurrences, the number of occurrences and localities can provide information on the abundance of a species in an area, though such exceptional fossilization potential is often restricted to marine environments [35]. Finally, more detailed information about the sampling localities of the fossil occurrences within each area, such as their distance from other areas, and the models incorporating ecological and climatic preferences and constraints may help understanding in more depth the dynamics of dispersals [26,29,64].

The framework presented here does not make explicit assumptions about the processes generating diversity and ignores the process of range inheritance. Previous research showed that it is possible to jointly estimate origination, dispersal and extinction rates from the fossil record [40]. However, this was done without explicitly accounting for sampling biases, and its application might be, therefore, restricted to well-sampled clades. Moreover, the estimation of origination rates requires the assumption that the lineages analysed form a monophyletic clade and are connected in a phylogenetic tree, though its topology may be unknown [40]. By contrast, the DES approach explicitly models the effects of incomplete sampling only and does not assume the lineages in a dataset to form a clade. These features make the model unable to infer origination processes and range inheritance, but they also make the method applicable to many empirical datasets with sparse fossil record and polyphyletic assemblages of taxa that have occurred in the same areas (such as our vascular plant dataset).

### (b) Cenozoic dispersals and extinctions in vascular plants

The analysis of the Cenozoic plant fossil record for the Northern Hemisphere demonstrates that biogeographic range evolution is not a time-homogeneous process, but varies through time and between areas. This represents a strong violation of the assumptions characterizing most phylogenetic methods in biogeography and involving constant or symmetric dispersal as well as constant or absent extinction (e.g. [11,20–22,65]). Our results show that such rate variation through time and space can be estimated from fossil data without incurring issues in parameter identifiability or convergence, provided that sufficient fossil occurrences are available. While we ran our analyses in a scenario with only two areas, further development and testing are necessary to assess the robustness of the approach for a larger number of areas.

The general temporal trends in extinction rates obtained under the DES model closely resemble those estimated under a birth–death model using global data [46]. Strikingly, the time slice characterized by a generally stable climate (32–14 Ma) also showed the lowest dispersal and extinction rates. By contrast, strong climate changes were associated with phases of high plant turnover with increased extinction rates and high dispersal rates. These results support previous findings suggesting that climate changes can foster waves of migration and dispersal [6,12,66–70].

The overall highest floristic interchange between North America and Eurasia was estimated in the most recent time slice (14–0 Ma). This phase of increased dispersal is unlikely to be linked with preservation biases because (i) the DES model is robust under a wide range of preservation rates (figure 3) and (ii) the estimated preservation rates through time do not suggest strong variations towards the present (figure 5; electronic supplementary material, figures S7 and S8). The factors driving the patterns observed remain unclear, but might be attributed to closer proximity between continents through the Bering Strait, increased land exposure during Pleistocene glacials and/or strong climatic oscillations that would have selected for taxa with higher dispersal ability and cold tolerance.

The shared biogeographic history of North America and Eurasia has been well studied (e.g. [71]) and connectivity among the flora and fauna across the Northern Hemisphere is suggested to be a relict of the Cenozoic (e.g. [72]). Our results further clarify Cenozoic patterns, showing high dispersal between continents in the Northern Hemisphere from 14 to 0 Ma, with slightly higher rates from North America to Eurasia. Higher asymmetry among dispersal rates is found during the Eocene cooling event, where dispersal rates from Eurasia are six times higher than those in the opposite direction (figure 5). Few previous studies have commented on the timing of dispersal among regions although Donoghue & Smith [72] detected increased migration out of Asia at different geological times, particularly over the last 30 Myr. In general, we show higher dispersal during epochs of global cooling (figure 6) with the highest dispersal out of Asia earlier during the Eocene and the highest dispersal out of North America during the most recent time interval. Based on meta-analyses of phylogenies Donoghue & Smith [72] showed that plants have many more North American–Asian disjunctions than animals, which is at odds with the large number of disjunctions in animals reported by Sanmartin *et al*. [10]. Analyses of plant and animal dispersal dynamics using the fossil record may help clarifying this discrepancy and further understand the historical biogeography of lineages in these areas.

### (c) The dispersal–extinction–sampling model and phylogeny-based historical biogeography

Unlike most phylogenetic methods, which have a strong focus on the estimation of ancestral ranges at the nodes of a tree, the main purpose of the DES model is investigating the anagenetic aspect of geographical range evolution by inferring dispersal and extinction rates across areas and through time. While it would be desirable to incorporate a cladogenetic component, as in the phylogenetic DEC model, within the DES framework, this addition would require the fossil lineages to be connected in a phylogenetic tree. Although phylogenetic inferences of fossil lineages with both extinct and extant lineages are possible (e.g. [32,73–76]), this option is limited to very few clades with exceptionally well sampled and studied fossil records. We emphasize, however, that the estimation of dispersal and extinction rates does not require known phylogenetic relationships among lineages, as demonstrated by our simulations. This is possible because dispersals and extinctions are assumed to occur independently along each lineage [18] and fossil occurrences provide serially (though incompletely) sampled biogeographic distributions. Finally, the focus on anagenetic processes allows us to analyse polyphyletic groups of organisms that shared similar geographical distributions and history and, although phylogenetically distant, can provide crucial information about the overall biotic interchange and connectivity between areas [11,13].

## 5. Prospects and conclusion

Incorporating fossil information in phylogeny-based biogeographic analyses is key to improving our estimates of ancestral ranges and their evolution [16,29,32,64]. The DES model contributes to available methods in macroevolution by inferring biogeographic rates using exclusively fossil data, without the need for a known phylogenetic tree and explicitly taking into account sampling biases. Both dispersal and extinction rates retrieved from DES are generally more accurate than those estimated under a similar model in a phylogenetic context [20,32]. The rates estimated from the (stratified) DES model might not be directly comparable with those obtained in a phylogenetic DEC analysis, e.g. due to different degrees of taxonomic resolution (genera versus species) or different taxonomic concepts. However, the relative variation between area-specific rates and through different time slices as estimated in DES analyses could be used to *inform* phylogeny-based analyses under DEC-like models. For instance, dispersal rates from a stratified DES analysis could provide a data-driven approach to define dispersal matrices in DEC analyses, as an alternative to subjective and typically untested dispersal contraints, with potentially strong effects on the resulting ancestral ranges [15,77].

Historical biogeography is experiencing a phase of substantial methodological advancement, with the development of complex and more realistic models that can improve our understanding of the spatio-temporal dynamics of taxa and their underlying mechanisms. We showed that dispersal and extinction of taxa can be confidently inferred from fossil data, once the sampling biases are accounted for in the model. Fossil-based estimates of dispersal and extinction rates, which also consider their asymmetries and temporal variation, can together reveal important trends in the biotic interchange and connectivity between areas. The analysis of fossil data provides evolutionary biologists with new opportunities to infer the dynamics of range evolution and diversification in deep time using information from both extinct and extant lineages.

## Supplementary Material

Supplementary figures and tables

## References

[1] Sepkoski JJ 1981 A factor analytic description of the Phanerozoic marine fossil record. Paleobiology 7, 36–53.

[2] Niklas KJ, Tiffney BH, Knoll AH 1983 Patterns in vascular land plant diversification. Nature 303, 614–616. (10.1038/303614a0)

[3] Raup DM 1986 Biological extinction in earth history. Science 231, 1528–1533. (10.1126/science.11542058) 11542058

[4] Van Valen L 1985 A theory of origination and extinction. Evol. Theory 7, 133–142.

[5] Gilinsky NL, Bambach RK 1987 Asymmetrical patterns of origination and extinction in higher taxa. Paleobiology 13, 427–445.

[6] Beard C 2002 Paleontology—east of Eden at the Paleocene/Eocene boundary. Science 295, 2028–2029. (10.1126/science.1070259) 11896261

[7] Alroy J 2009 Speciation and extinction in the fossil record of North American mammals, pp. 301–323. Cambridge, UK: Cambridge University Press.

[8] Antonelli A, Sanmartín I 2011 Why are there so many plant species in the Neotropics? Taxon 60, 403–414.

[9] Hoorn C, Mosbrugger V, Mulch A, Antonelli A 2013 Biodiversity from mountain building. Nat. Geosci. 6, 154 (10.1038/ngeo1742)

[10] Sanmartín I, Enghoff H, Ronquist F 2001 Patterns of animal dispersal, vicariance and diversification in the Holarctic. Biol. J. Linn. Soc. 73, 345–390. (10.1111/j.1095-8312.2001.tb01368.x)

[11] Sanmartín I, Van Der Mark P, Ronquist F 2008 Inferring dispersal: a Bayesian approach to phylogeny-based island biogeography, with special reference to the Canary Islands. J. Biogeogr. 35, 428–449. (10.1111/j.1365-2699.2008.01885.x)

[12] Antonelli A, Zizka A, Silvestro D, Scharn R, Cascales-Miñana B, Bacon CD 2015 An engine for global plant diversity: highest evolutionary turnover and emigration in the American tropics. Front. Genet. 6, 201500130 (10.3389/fgene.2015.00130) PMC4389561 25904934

[13] Bacon CD, Silvestro D, Jaramillo CA, Smith BT, Chakrabarty P, Antonelli A 2015 Biological evidence supports an early and complex emergence of the Isthmus of Panama. Proc. Natl Acad. Sci. USA 112, 428–449. (10.1073/pnas.1423853112) PMC4434730 25918375

[14] Mairal M, Pokorny L, Aldasoro JJ, Alarcon M, Sanmartin I 2015 Ancient vicariance and climate-driven extinction explain continental-wide disjunctions in Africa: the case of the Rand Flora genus *Canarina* (Campanulaceae). Mol. Ecol. 24, 1335–1354. (10.1111/mec.13114) 25688489

[15] Ree RH, Sanmartín I 2009 Prospects and challenges for parametric models in historical biogeographical inference. J. Biogeogr. 36, 1211–1220. (10.1111/j.1365-2699.2008.02068.x)

[16] Crisp MD, Trewick SA, Cook LG 2011 Hypothesis testing in biogeography. Trends Ecol. Evol. 26, 66–72. (10.1016/j.tree.2010.11.005) 21146898

[17] Ronquist F 1997 Dispersal-vicariance analysis: a new approach to the quantification of historical biogeography. Syst. Biol. 46, 195–203. (10.1093/sysbio/46.1.195)

[18] Ree RH, Moore BR, Webb CO, Donoghue MJ 2005 A likelihood framework for inferring the evolution of geographic range on phylogenetic trees. Evolution 59, 2299–2311. (10.1111/j.0014-3820.2005.tb00940.x) 16396171

[19] Nylander JAA, Olsson U, Alström P, Sanmartín I 2008 Accounting for phylogenetic uncertainty in biogeography: a Bayesian approach to dispersal-vicariance analysis of the thrushes (Aves: *Turdus*). Syst. Biol. 57, 257–268. (10.1080/10635150802044003) 18425716

[20] Ree RH, Smith S 2008 Maximum likelihood inference of geographic range evolution by dispersal, local extinction, and cladogenesis. Syst. Biol. 57, 4–14. (10.1080/10635150701883881) 18253896

[21] Landis MJ, Matzke NJ, Moore BR, Huelsenbeck JP 2013 Bayesian analysis of biogeography when the number of areas is large. Syst. Biol. 62, 789–804. (10.1093/sysbio/syt040) 23736102 PMC4064008

[22] Matzke NJ 2014 Model selection in historical biogeography reveals that founder-event speciation is a crucial process in island clades. Syst. Biol. 63, 951–970. (10.1093/sysbio/syu056) 25123369

[23] Matzke NJ 2013 Probabilistic historical biogeography: new models for founder-event speciation, imperfect detection, and fossils allow improved accuracy and model-testing. Front. Biogeogr. 5, 242–248.

[24] Smith SA 2009 Taking into account phylogenetic and divergence-time uncertainty in a parametric biogeographical analysis of the Northern Hemisphere plant clade Caprifolieae. J. Biogeogr. 36, 2324–2337. (10.1111/j.1365-2699.2009.02160.x)

[25] Bielejec F, Lemey P, Baele G, Rambaut A, Suchard MA 2014 Inferring heterogeneous evolutionary processes through time: from sequence substitution to phylogeography. Syst. Biol. 63, 493–504. (10.1093/sysbio/syu015) 24627184 PMC4055869

[26] Lawing AM, Matzke NJ 2014 Conservation paleobiology needs phylogenetic methods. Ecography 37, 1109–1122. (10.1111/ecog.00783)

[27] Goldberg E, Lancaster L, Ree R 2011 Phylogenetic inference of reciprocal effects between geographic range evolution and diversification. Syst. Biol. 60, 451–465. (10.1093/sysbio/syr046) 21551125

[28] Fitzjohn RG 2012 Diversitree: comparative phylogenetic analyses of diversification in R. Methods Ecol. Evol. 3, 1084–1092. (10.1111/j.2041-210X.2012.00234.x)

[29] Meseguer AS, Lobo JM, Ree R, Beerling DJ, Sanmartin I 2015 Integrating fossils, phylogenies, and niche models into biogeography to reveal ancient evolutionary history: the case of *Hypericum* (Hypericaceae). Syst. Biol. 64, 215–232. (10.1093/sysbio/syu088) 25398444 PMC4380036

[30] Rabosky DL, Goldberg EE 2015 Model inadequacy and mistaken inferences of trait-dependent speciation. Syst. Biol. 64, 340–355. (10.1093/sysbio/syu131) 25601943

[31] Davis MP, Midford PE, Maddison W 2013 Exploring power and parameter estimation of the BiSSE method for analyzing species diversification. BMC Evol. Biol. 13, 38 (10.1186/1471-2148-13-38) 23398853 PMC3583807

[32] Wood HM, Matzke NJ, Gillespie RG, Griswold CE 2013 Treating fossils as terminal taxa in divergence time estimation reveals ancient vicariance patterns in the palpimanoid spiders. Syst. Biol. 62, 264–284. (10.1093/sysbio/sys092) 23192838

[33] Python Software Foundation. 2013 Python language reference, version 2.7. Available at http://www.python.org

[34] Silvestro D, Salamin N, Schnitzler J 2014 PyRate: a new program to estimate speciation and extinction rates from incomplete fossil record. Methods Ecol. Evol. 5, 1126–1131. (10.1111/2041-210X.12263)

[35] Liow LH, Stenseth NC 2007 The rise and fall of species: implications for macroevolutionary and macroecological studies. Proc. R. Soc. B 274, 2745–2752. (10.1098/rspb.2007.1006) PMC2279224 17711843

[36] Torres V, Hooghiemstra H, Lourens L, Tzedakis PC 2013 Astronomical tuning of long pollen records reveals the dynamic history of montane biomes and lake levels in the tropical high Andes during the Quaternary. Quat. Sci. Rev. 63, 59–72. (10.1016/j.quascirev.2012.11.004)

[37] Foote M 2001 Inferring temporal patterns of preservation, origination, and extinction from taxonomic survivorship analysis. Paleobiology 27, 602–630. (10.1666/0094-8373(2001)027<0602:ITPOPO>2.0.CO;2)

[38] Quental TB, Marshall CR 2013 How the Red Queen drives terrestrial mammals to extinction. Science 341, 290–292. (10.1126/science.1239431) 23788731

[39] Silvestro D, Schnitzler J, Liow LH, Antonelli A, Salamin N 2014 Bayesian estimation of speciation and extinction from incomplete fossil occurrence data. Syst. Biol. 63, 349–367. (10.1093/sysbio/syu006) 24510972 PMC4361715

[40] Goldberg EE, Roy K, Lande R, Jablonski D 2005 Diversity, endemism, and age distributions in macroevolutionary sources and sinks. Am. Nat. 165, 623–633. (10.1086/430012) 15937743

[41] Ronquist F, Huelsenbeck JP, Van Der Mark P, Lemey P 2007 Bayesian phylogenetic analysis using MrBayes. In The phylogenetic handbook (eds P Lemey, M Salemi, A-M Vandamme), pp. 1–63. Cambridge, UK: Cambridge University Press.

[42] Metropolis N, Rosenbluth AW, Rosenbluth MN, Teller AW, Teller E 1953 Equations of state calculations by fast computing machines. J. Chem. Phys. 21, 1087–1091. (10.1063/1.1699114)

[43] Hastings WK 1970 Monte Carlo sampling methods using Markov chains and their applications. Biometrika 57, 97–109. (10.1093/biomet/57.1.97)

[44] Rambaut A, Suchard MA, Xie D, Drummond AJ 2014 Tracer v1.6. See http://beast.bio.ed.ac.uk/tracer.

[45] Silvestro D, Schnitzler J, Zizka G 2011 A Bayesian framework to estimate diversification rates and their variation through time and space. BMC Evol. Biol. 11, 311 (10.1186/1471-2148-11-311) 22013891 PMC3224121

[46] Silvestro D, Cascales-Miñana B, Bacon CD, Antonelli A 2015 Revisiting the origin and diversification of vascular plants through a comprehensive Bayesian analysis of the fossil record. New Phytol. 207, 425–436. (10.1111/nph.13247) 25619401 PMC4949670

[47] Lartillot N, Philippe H 2006 Computing Bayes factors using thermodynamic integration. Syst. Biol. 55, 195–207. (10.1080/10635150500433722) 16522570

[48] Xie W, Lewis PO, Fan Y, Kuo L, Chen M-H 2011 Improving marginal likelihood estimation for Bayesian phylogenetic model selection. Syst. Biol. 60, 150–160. (10.1093/sysbio/syq085) 21187451 PMC3038348

[49] Baele G, Lemey P, Bedford T, Rambaut A, Suchard M, Alekseyenko A 2012 Improving the accuracy of demographic and molecular clock model comparison while accommodating phylogenetic uncertainty. Mol. Biol. Evol. 29, 2157–2167. (10.1093/molbev/mss084) 22403239 PMC3424409

[50] Kass RE, Raftery AE 1995 Bayes factors. J. Am. Stat. Assoc. 90, 773–795. (10.1080/01621459.1995.10476572)

[51] Xing Y, Onstein RE, Carter RJ, Stadler T, Linder HP 2014 Fossils and a large molecular phylogeny show that the evolution of species richness, generic diversity and turnover rates are disconnected. Evolution 68, 2821–2832. (10.1111/evo.12489) 25041629

[52] WCSP. *World checklist of selected plant families*. http://apps.kew.org/wcsp/ (accessed May 2015).

[53] USDA NRCS. *The PLANTS database*. http://plants.usda.gov (accessed May 2015).

[54] *Flora Mesoamericana*. http://www.tropicos.org/Project/FM (accessed May 2015).

[55] *Euro+Med PlantBase. The information resource for Euro-Mediterranean plant diversity*. http://ww2.bgbm.org/EuroPlusMed/ (accessed May 2015).

[56] Bundesamt für Naturschutz. *FloraWeb*. http://www.floraweb.de/ (accessed May 2015).

[57] Mossberg B, Stenberg L 2010 *Den nya nordiska Floran*. Stockholm, Sweden: Bonnier Fakta.

[58] eFloras. *Flora of China*. http://www.efloras.org/flora_page.aspx?flora_id=2 (accessed May 2015).

[59] Iwatsuki K, Yamazaki T, Boufford DE, Ohba H 2005 *Flora of Japan*. Tokyo, Japan: Kodansha

[60] GBIF. 2014 *Global Biodiversity Information Facility—free and open access to biodiveristy data*. http://www.gbif.org (accessed May 2015).

[61] Zachos JC, Dickens GR, Zeebe RE 2008 An early Cenozoic perspective on greenhouse warming and carbon-cycle dynamics. Nature 451, 279–283. (10.1038/nature06588) 18202643

[62] Silvestro D, Antonelli A, Salamin N, Quental TB 2015 The role of clade competition in the diversification of North American canids. Proc. Natl Acad. Sci. USA 112, 8684–8689. (10.1073/pnas.1502803112) 26124128 PMC4507235

[63] Yang Z 1994 Maximum likelihood phylogenetic estimation from DNA sequences with variable rates over sites: approximate methods. J. Mol. Evol. 39, 306–314. (10.1007/BF00160154) 7932792

[64] Metcalf JL, Prost S, Nogués-Bravo D, DeChaine EG, Anderson C, Batra P, Araújo MB, Cooper A, Guralnick RP 2014 Integrating multiple lines of evidence into historical biogeography hypothesis testing: a Bison case study. Proc. R. Soc. B 281, 20132782 (10.1098/rspb.2013.2782) PMC3896022 24403338

[65] Yu Y, Harris AJ, Blair C, He X 2015 {RASP} (reconstruct ancestral state in phylogenies): a tool for historical biogeography. Mol. Phylogenet. Evol. 87, 46–49. (10.1016/j.ympev.2015.03.008) 25819445

[66] Beard C 1998 East of Eden: Asia as an important center of taxonomic origination in mammalian evolution. Bull. Carnegie Mus. Nat. Hist. 34, 5–39.

[67] Taberlet P, Fumagalli L, Wust-Saucy AG, Cosson JF 1998 Comparative phylogeography and postglacial colonization routes in Europe. Mol. Ecol. 7, 453–464. (10.1046/j.1365-294x.1998.00289.x) 9628000

[68] Bowen GJ, Clyde WC, Koch PL, Ting SY, Alroy J, Tsubamoto T, Wang YQ, Wang Y 2002 Mammalian dispersal at the Paleocene/Eocene boundary. Science 295, 2062–2065. (10.1126/science.1068700) 11896275

[69] Lieberman BS 2003 Paleobiogeography: the relevance of fossils to biogeography. Ann. Rev. Ecol. Evol. Syst. 34, 51–69. (10.1146/annurev.ecolsys.34.121101.153549)

[70] Antonelli A, Verola CF, Parisod C, Gustafsson ALS 2010 Climate cooling promoted the expansion and radiation of a threatened group of South American orchids (Epidendroideae: Laeliinae). Biol. J. Linn. Soc. 100, 597–607. (10.1111/j.1095-8312.2010.01438.x)

[71] Wen J, Ickert-Bond S, Nie Z-L, Li R 2010 Timing and modes of evolution of eastern Asian-North American biogeographic disjunctions in seed plants. In *Darwin's Heritage Today: Proc. of the Darwin 2010 Beijing Int. Conf.* (eds M Long, HY Gu, ZH Zhou), pp. 252–269. Beijing, China: Higher Education Press.

[72] Donoghue MJ, Smith SA 2004 Patterns in the assembly of temperate forests around the Northern Hemisphere. Phil. Trans. R. Soc. Lond. B 359, 1633–1644. (10.1098/rstb.2004.1538) 15519978 PMC1693435

[73] Bapst DW 2012 paleotree: an R package for paleontological and phylogenetic analyses of evolution. Methods Ecol. Evol. 3, 803–807. (10.1111/j.2041-210X.2012.00223.x)

[74] Ronquist F, Klopfstein S, Vilhelmsen L, Schulmeister S, Murray DL, Rasnitsyn AP 2012 A total-evidence approach to dating with fossils, applied to the early radiation of the Hymenoptera. *Syst. Biol.* 61, 973–999. (10.1093/sysbio/sys058) PMC3478566 22723471

[75] Heath TA, Hulsenbeck JP, Stadler T 2014 The fossilized birth-death process for coherent calibration of divergence-time estimates. Proc. Natl Acad. Sci. USA 111, 2957–2966. (10.1073/pnas.1319091111) PMC4115571 25009181

[76] Slater GJ 2015 Iterative adaptive radiations of fossil canids show no evidence for diversity-dependent trait evolution. Proc. Natl Acad. Sci. USA 112, 4897–4902. (10.1073/pnas.1403666111) 25901311 PMC4413353

[77] Chacón J, Renner SS 2014 Assessing model sensitivity in ancestral area reconstruction using Lagrange: a case study using the Colchicaceae family. J. Biogeogr. 41, 1414–1427. (10.1111/jbi.12301)

