# Supplementary material for: Fossil biogeography: a new model to infer dispersal, extinction and sampling from palaeontological data
Source: Philos Trans R Soc Lond B Biol Sci. 2016 Apr 5;371(1691):20150225. doi: 10.1098/rstb.2015.0225 (PMC4810818; doi:10.1098/rstb.2015.0225)
Supplement: Supplementary figures and tables [file rstb20150225supp1.pdf]

Version dated: January 13, 2016

Fossil Biogeography: A new model to infer dispersal, extinction and sampling from paleontological data

Daniele Silvestro[1,2], Alexander Zizka[1], Christine D. Bacon,[1], Borja Cascales-Miñana[3], Nicolas Salamin[2,4] and Alexandre Antonelli[1,5]

[1] *Department of Biological and Environmental Sciences, University of Gothenburg, Carl Skottsbergs gata 22B, 413 19 Gothenburg, Sweden;*

[2] *Department of Ecology and Evolution, University of Lausanne, 1015 Lausanne, Switzerland;*

[3] *Department de Geology, University of Liege, Belgium;*

[4] *Swiss Institute of Bioinformatics, Quartier Sorge, 1015 Lausanne, Switzerland;*

[5] *Gothenburg Botanical Garden, Carl Skottsbergs gata 22A, 413 19 Gothenburg, Sweden*

Corresponding author: Daniele Silvestro: silvestro.daniele@unil.ch.

Supplementary Figures

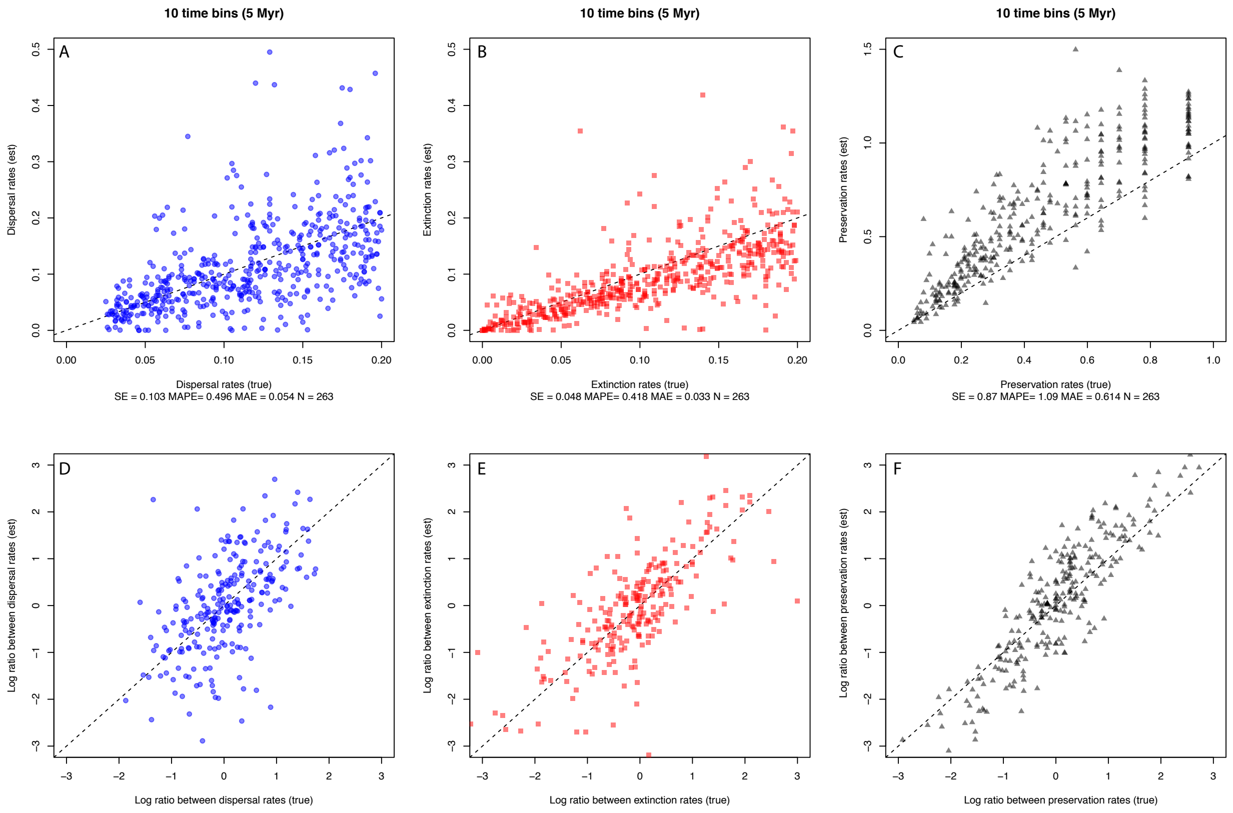

Figure S1: Dispersal, extinction, and preservation rates obtained from simulations, using time bins of 5 Myr (A-C). True rates (used to simulate the data) are plotted against estimated rates (maximum a posteriori). Points below the diagonal (dashed line) represent underestimates, points above the diagonal represent overestimates. The ability of the model to recover rate asymmetry is shown by plotting the log ratio between the true rates against the log ratio between estimated rates (D-F).

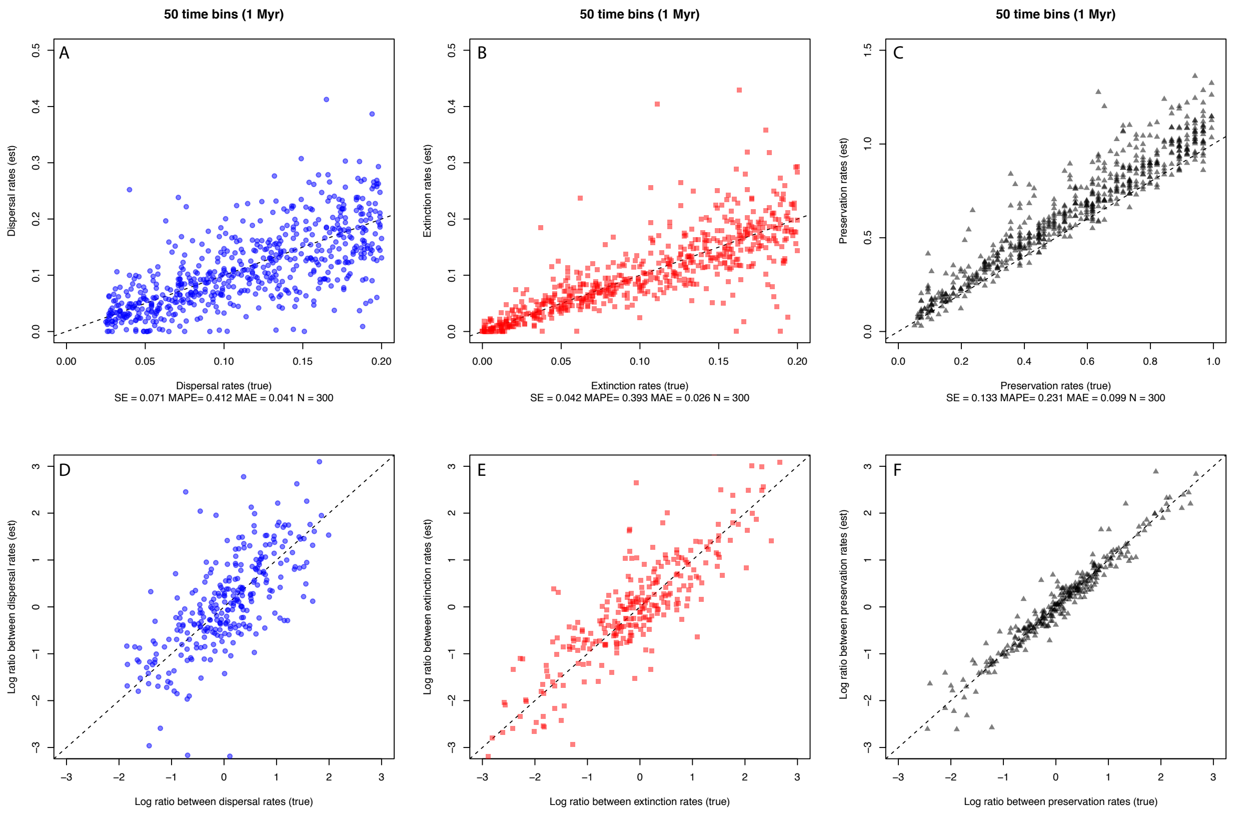

Figure S2: Dispersal, extinction, and preservation rates obtained from simulations, using time bins of 1 Myr (A-C). True rates (used to simulate the data) are plotted against estimated rates (maximum a posteriori). Points below the diagonal (dashed line) represent underestimates, points above the diagonal represent overestimates. The ability of the model to recover rate asymmetry is shown by plotting the log ratio between the true rates against the log ratio between estimated rates (D-F).

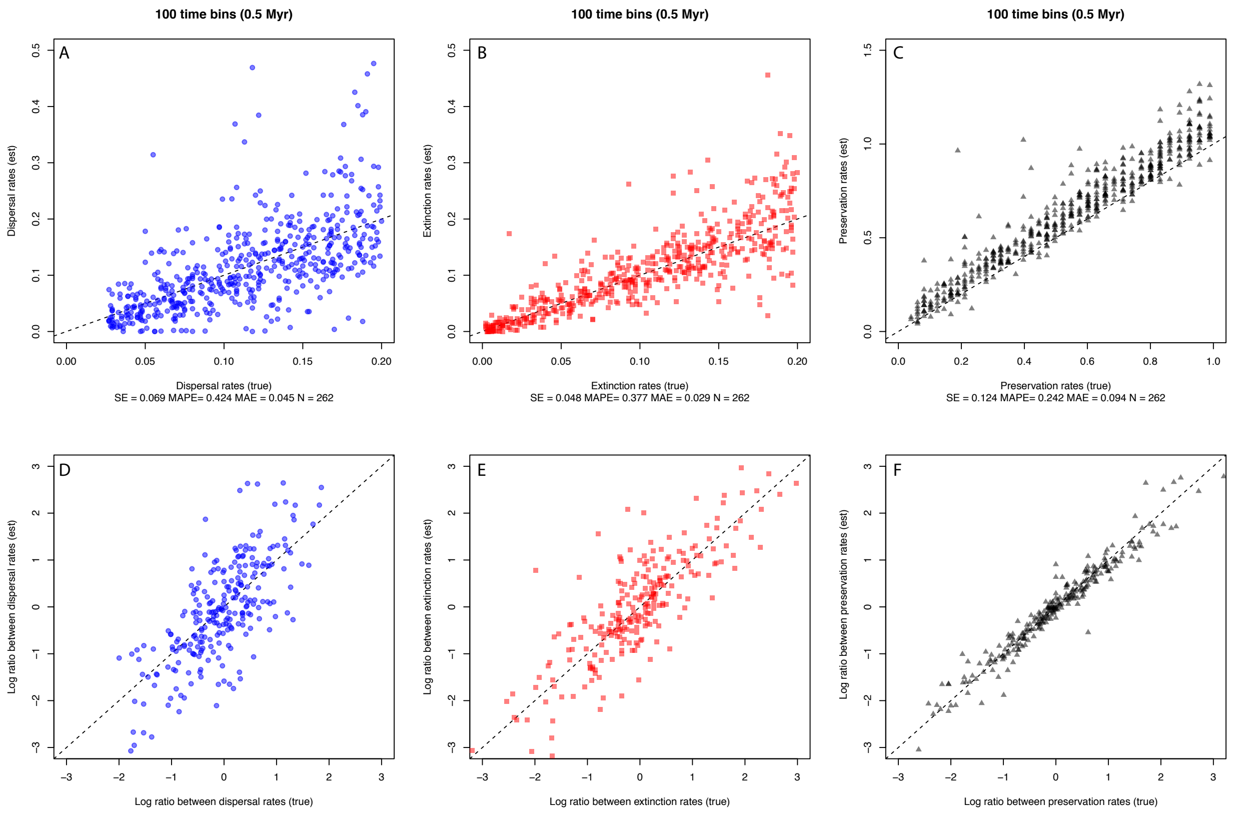

Figure S3: Dispersal, extinction, and preservation rates obtained from simulations, using time bins of 0.5 Myr (A-C). True rates (used to simulate the data) are plotted against estimated rates (maximum a posteriori). Points below the diagonal (dashed line) represent underestimates, points above the diagonal represent overestimates. The ability of the model to recover rate asymmetry is shown by plotting the log ratio between the true rates against the log ratio between estimated rates (D-F).

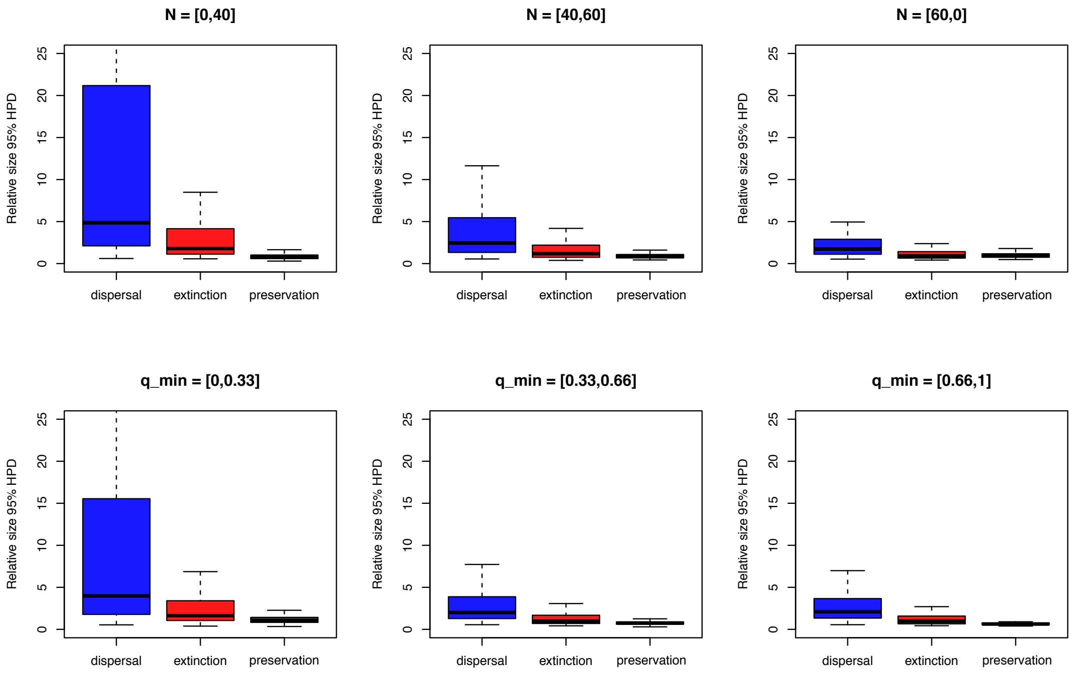

Figure S4: Relative size of the credible intervals around the estimated dispersal, extinction, and preservation rates. Estimates are based on time bins of 5 Myr to code the fossil geographic ranges.

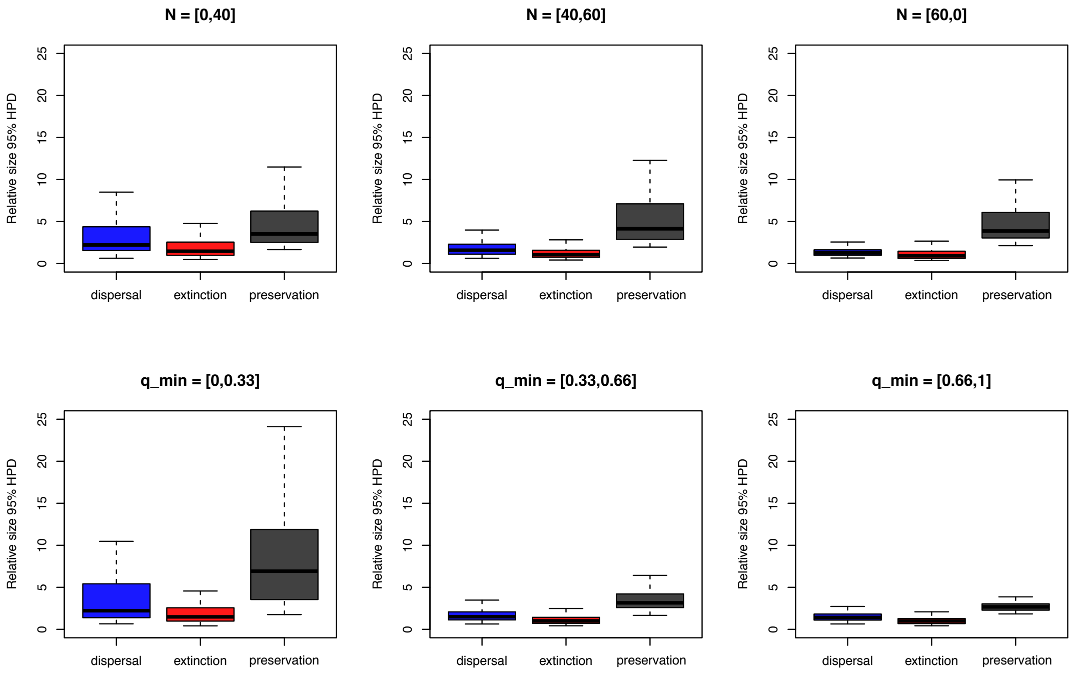

Figure S5: Relative size of the credible intervals around the estimated dispersal, extinction, and preservation rates. Estimates are based on time bins of 1 Myr to code the fossil geographic ranges.

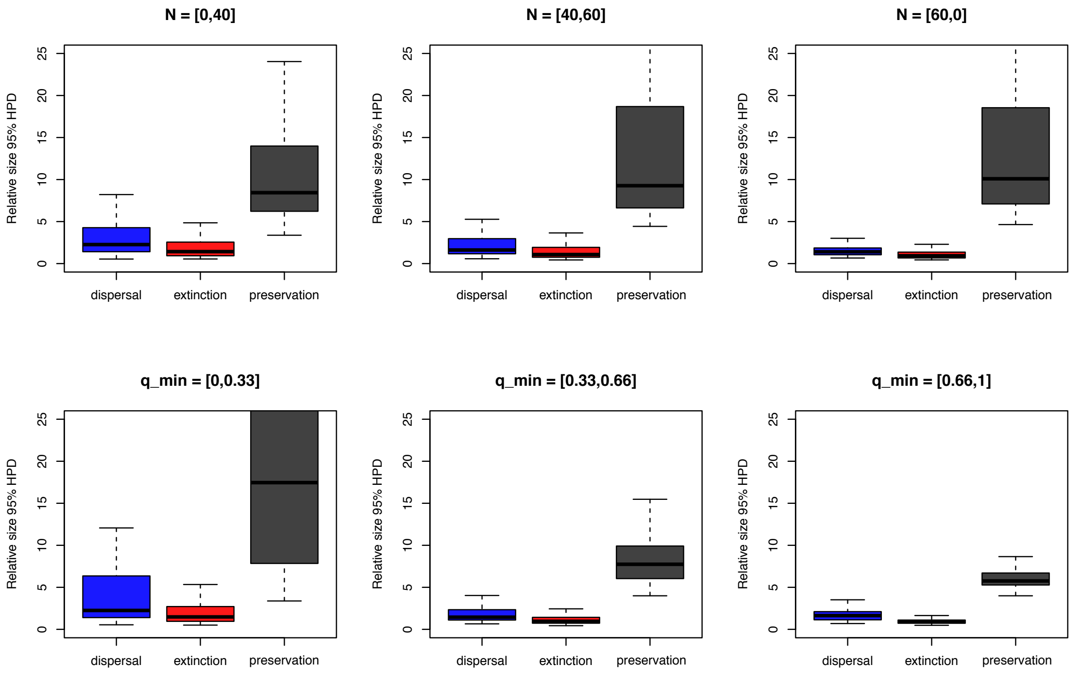

Figure S6: Relative size of the credible intervals around the estimated dispersal, extinction, and preservation rates. Estimates are based on time bins of 0.5 Myr to code the fossil geographic ranges.

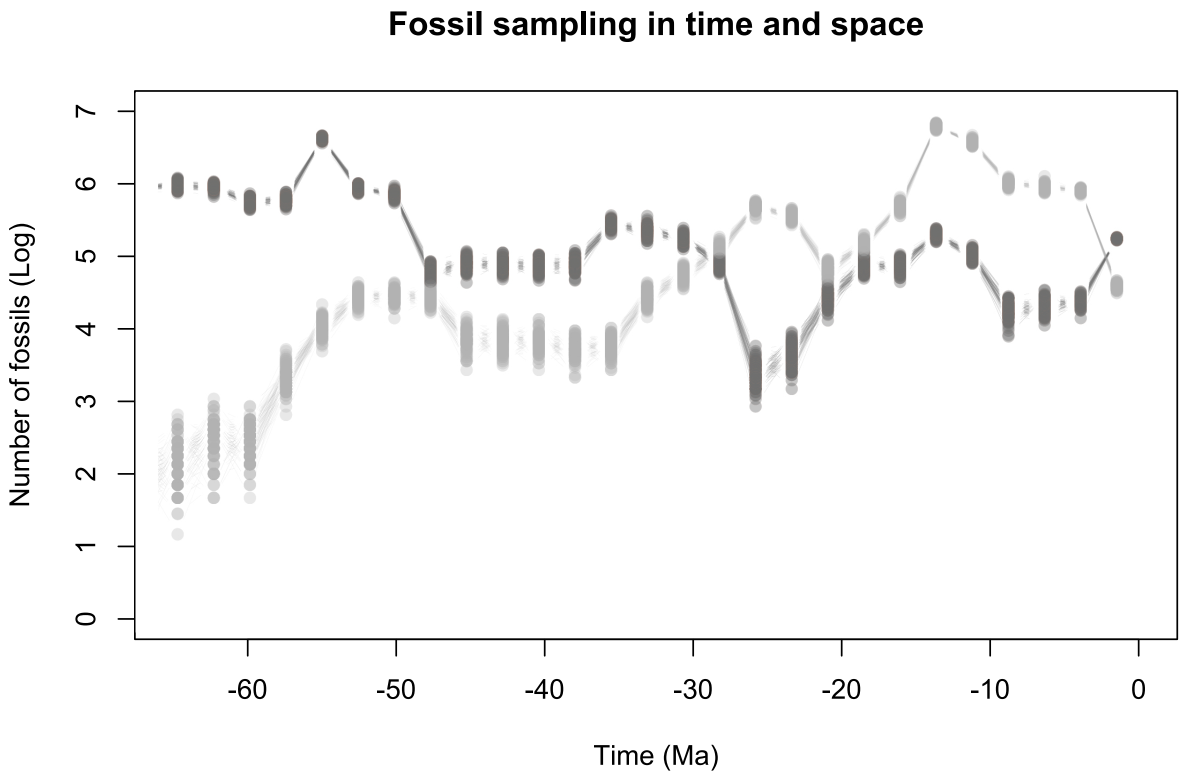

Figure S7: Number of vascular plant fossil occurrences sampled in North America (dark grey) and Eurasia (light grey) throughout the Cenozoic counted within 2.5 Myr time bins. Counts were repeated over 100 randomized data sets showing the effect of uncertainties in the age of fossil occurrences (see Methods).

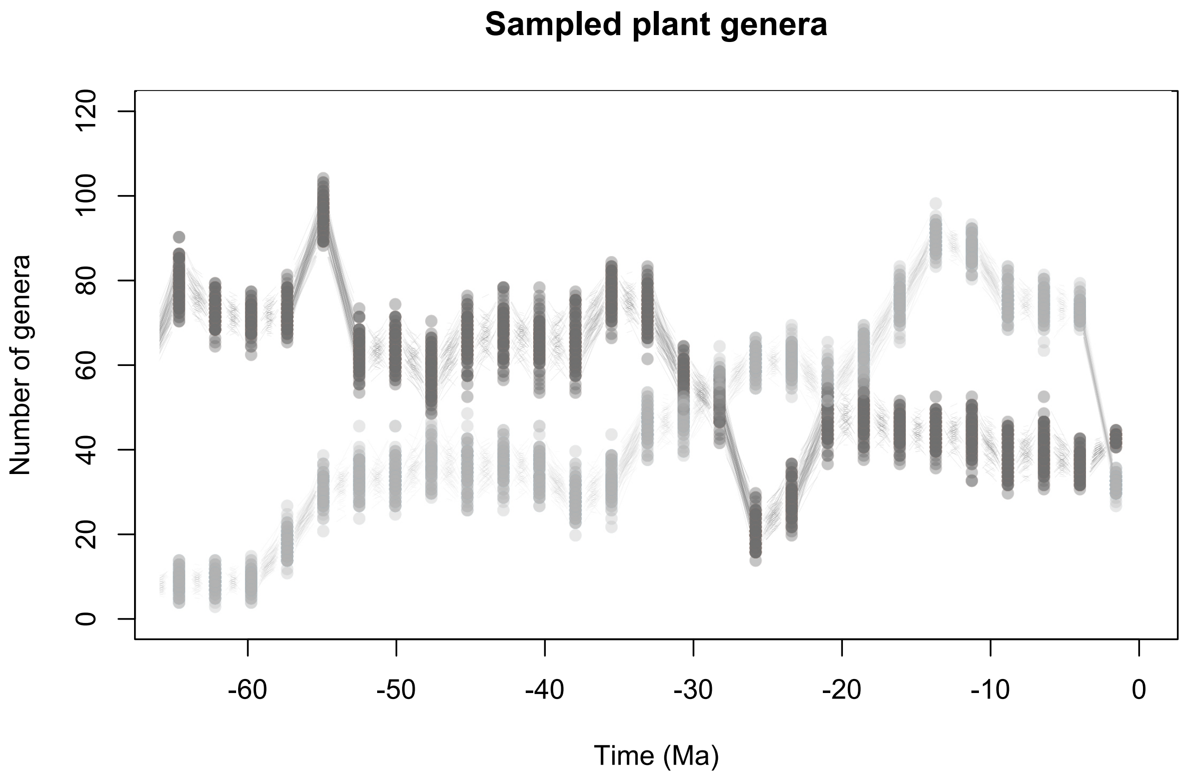

Figure S8: Number of sampled genera of vascular plants in North America (dark grey) and Eurasia (light grey) throughout the Cenozoic counted within 2.5 Myr time bins. Counts were repeated over 100 randomized data sets showing the effect of uncertainties in the age of fossil occurrences (see Methods).

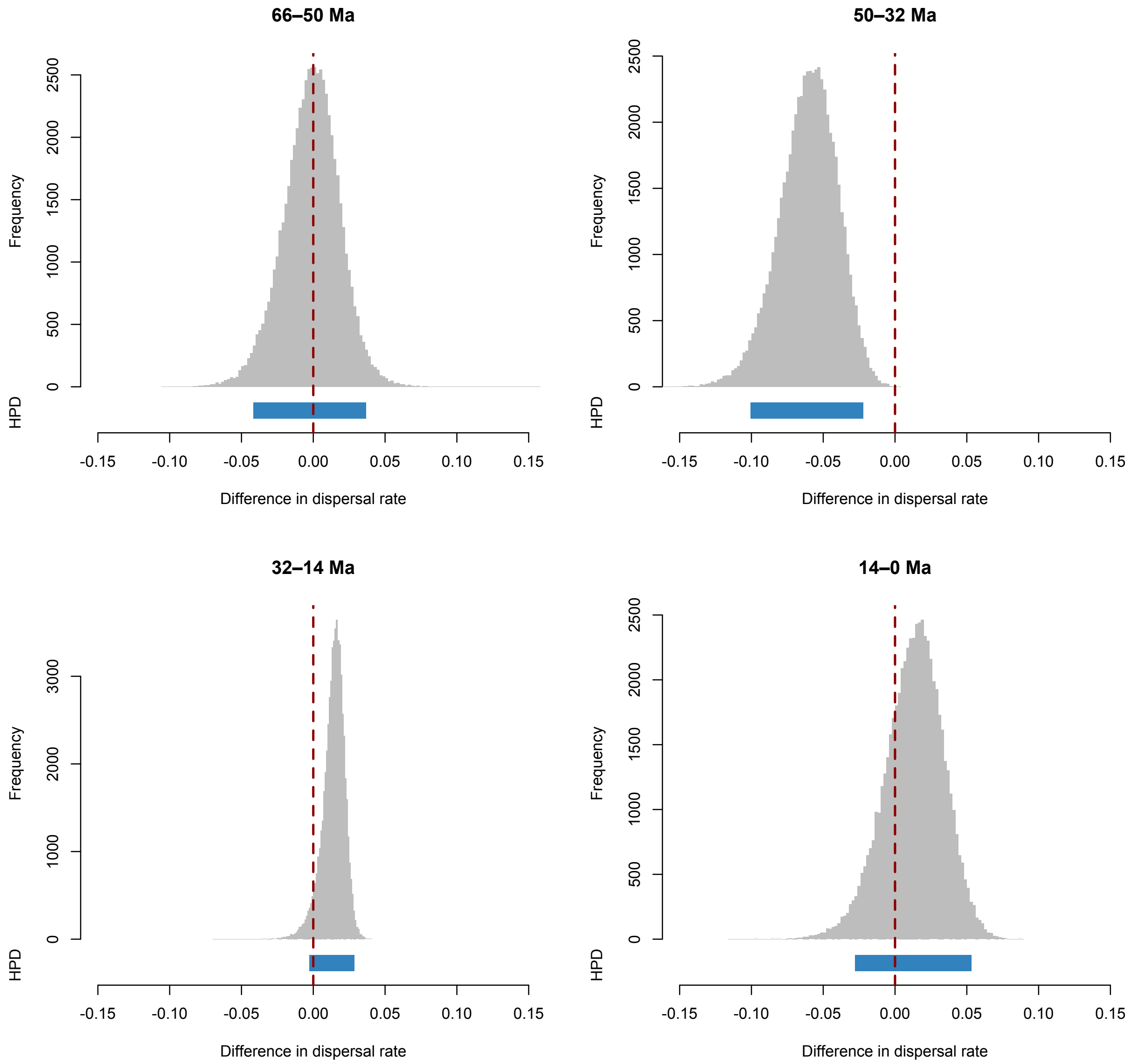

Figure S9: Asymmetry in dispersal rates through time. Histograms show the difference between dispersal rates calculated as $d_{NA->EA} - d_{EA->NA}$ from all posterior samples obtained through MCMC. Thus positive values indicate a higher dispersal rate from North America to Eurasia and negative values indicate higher dispersal rate from Eurasia to North America. Blue bars indicate the 95% credible intervals. Rate asymmetry is considered as significant when 0 falls outside of the 95% credible intervals.

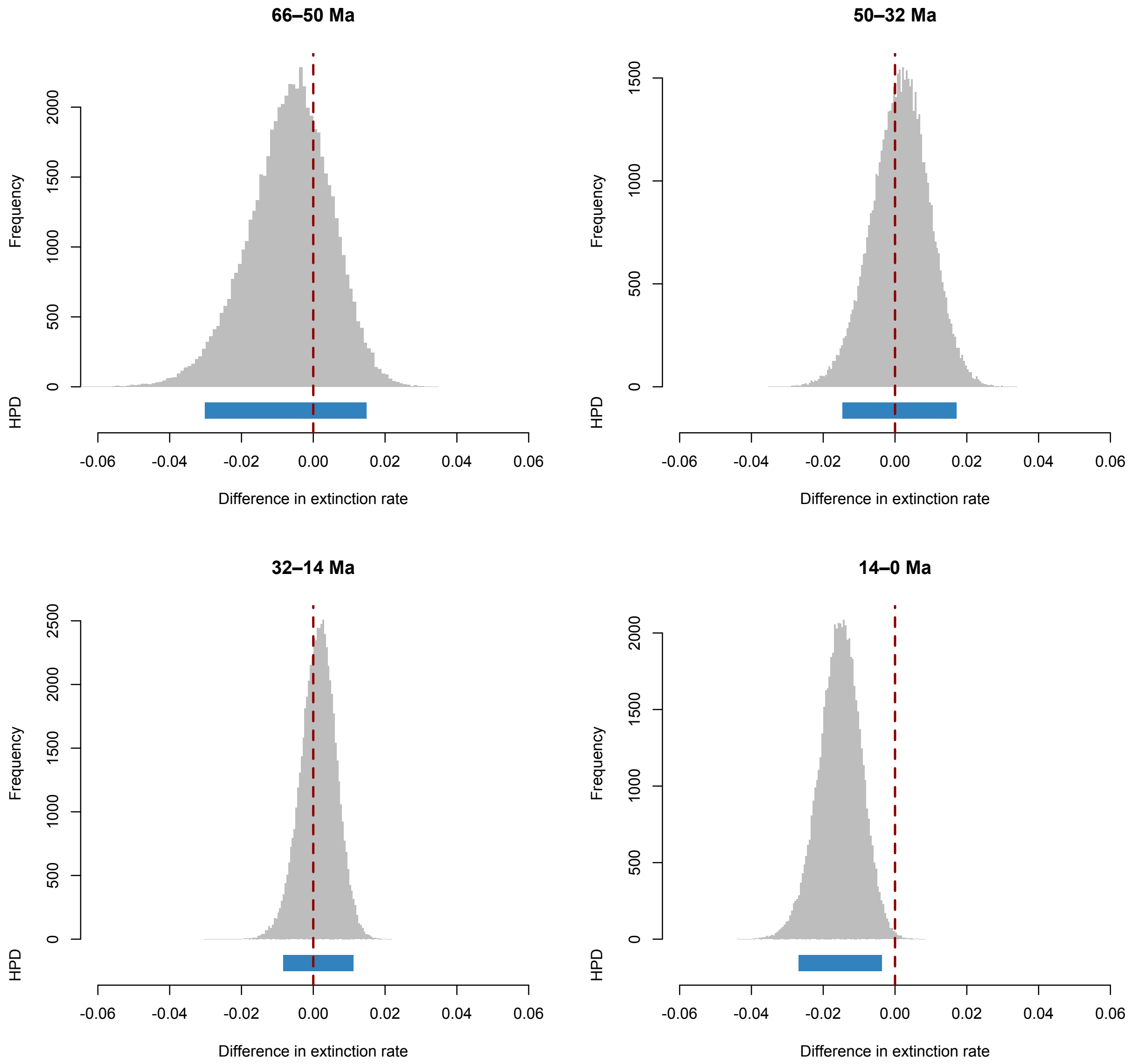

Figure S10: Asymmetry in dispersal rates through time. Histograms show the difference between local extinction rates calculated as $e_{NA} - e_{EA}$ from all posterior samples obtained through MCMC. Thus positive values indicate a higher extinction rate in North America and negative values indicate higher extinction rate in Eurasia. Blue bars indicate the 95% credible intervals. Rate asymmetry is considered as significant when 0 falls outside of the 95% credible intervals.

Table S1: Summary of the Cenozoic plant fossil data set (continues in next pages).

Genus	No. occurrences	
	NA	EA
Abies	57	32
Acacia	2	0
Acalypha	1	1
Acer	141	147
Acrovena	1	0
Ailanthus	8	18
Alangium	5	8
Alisma	0	33
Alisporites	0	1
Alnus	166	173
Amelanchier	23	0
Amelanchites	5	0
Ampelopsis	112	62
Andromeda	0	6
Anemia	18	4
Aphananthe	1	5
Aralia	2	58
Araucaria	4	0
Araucariacites	0	1
Araucarioxylon	0	1
Araucarites	0	8
Arbutus	14	0
Arctostaphylos	5	0
Artocarpus	0	1
Arundo	2	0
Asclepiadites	1	0
Asplenium	5	1
Athrotaxis	0	22
Averrhoites	89	0
Azolla	23	104
Azollopsis	9	0
Baculatisporites	0	2
Balmeisporites	2	0
Beaupreaidites	0	1
Berchemia	2	10
Berhamniphyllum	3	0
Betula	87	87
Blechnum	4	0
Bombacacidites	31	3
Bursericarpum	2	5
Calycites	15	0

Table S2: Summary of the Cenozoic plant fossil data set (continued).

Genus	No. occurrences	
	NA	EA
Calyptranthes	1	0
Camelliacarpoidea	0	1
Canariophyllum	1	0
Canarium	12	1
Carpinus	3	122
Carpolithes	3	7
Carya	138	68
Castanea	28	14
Castanopsis	15	14
Cayratia	0	1
Ceanothus	13	0
Cedrela	24	2
Cedrelospermum	24	1
Celastrus	9	0
Celtis	60	92
Ceratozamia	0	2
Cercidiphyllum	172	15
Cercis	17	1
Chaetoptelea	36	0
Cicatricosisporites	51	4
Cinnamomum	19	53
Cissus	41	0
Cladrastis	24	22
Classopollis	0	2
Cleyera	2	13
Cocculus	21	6
Comptonia	8	32
Cornus	53	66
Corydalis	0	1
Corylopsis	0	20
Corylus	33	26
Cranea	5	0
Crassivitisemen	0	1
Crataegus	34	52
Cruciptera	3	0
Cupania	4	0
Cyathea	19	0
Cycadeospermum	0	9
Cyclopteris	1	0
Cymodocea	2	0
Cyperacites	6	0

Table S3: Summary of the Cenozoic plant fossil data set (continued).

Genus	No. occurrences	
	NA	EA
Cyperus	3	10
Dalbergia	9	0
Decodon	3	145
Dennstaedtia	1	0
Desmodium	0	1
Dicotylophyllum	48	19
Dillenites	4	0
Diospyros	7	8
Diploclisia	1	2
Dipteronia	10	0
Dombeya	53	0
Dryophyllum	13	8
Dryopteris	23	0
Elaeodendron	0	1
Engelhardtia	12	8
Equisetum	89	3
Euphorbiotheca	0	8
Exbucklandia	2	0
Fagus	7	107
Ficus	86	26
Firmianites	1	0
Fokienia	3	0
Fraxinus	40	15
Gleditsia	2	10
Gleichenia	3	0
Gleicheniidites	52	2
Glyptostrobus	164	120
Gordonia	10	4
Guettarda	1	0
Halesia	3	37
Halodule	3	0
Hamamelites	7	0
Hemitelia	2	0
Heyderia	1	0
Hicoria	1	0
Holmskioldia	1	0
Hooleya	0	1
Hydrangea	14	4
Hydrocharis	0	13
Icacinicarya	0	22
Inga	3	0

Table S4: Summary of the Cenozoic plant fossil data set (continued).

Genus	No. occurrences	
	NA	EA
Isoetites	13	0
Juglans	21	36
Juniperus	12	4
Kalmia	3	2
Karinschmidtia	0	1
Klukisporites	0	1
Koelreuteria	14	2
Lannea	0	1
Larix	19	1
Laurocarpum	3	62
Laurophyllum	24	23
Laurus	24	4
Leguminosites	14	7
Lemnaceae	5	0
Limnobiophyllum	0	1
Liquidambar	13	92
Litsea	0	12
Lygodium	61	12
Lyonia	1	5
Magnolia	51	161
Mahonia	60	2
Malapoenna	1	0
Mallotus	9	5
Marsilea	0	10
Martinmuellera	0	1
Meliosma	32	42
Menispermites	24	0
Metasequoia	144	39
Micropodium	1	0
Minerisporites	1	6
Morus	1	28
Musophyllum	3	0
Myrica	8	157
Myristicacarpum	0	2
Natsiatum	0	7
Nelumbago	10	0
Nelumbium	3	0
Nelumbo	13	3
Neolitsea	0	4
Nerium	1	0
Nymphaea	3	22

Table S5: Summary of the Cenozoic plant fossil data set (continued).

Genus	No. occurrences	
	NA	EA
Nyssa	36	119
Nyssidium	1	0
Ocotea	28	5
Onoclea	19	0
Osmunda	20	13
Ostrya	10	45
Palaeocarya	7	0
Palaeophytocrene	6	4
Palaeosinomenium	2	9
Palaeovitis	0	2
Paliurus	7	37
Palmacites	1	0
Palmocarpon	7	0
Parabaena	0	5
Paranymphaea	45	0
Paraphyllanthoxylon	3	0
Paraternstroemia	2	0
Parthenocissus	6	23
Pecopteris	0	1
Penosphyllum	15	0
Pentoperculum	1	1
Pericampylus	0	1
Persea	16	24
Persites	58	0
Philadelphus	1	0
Phragmites	4	18
Phyllites	20	0
Physalis	0	11
Phytocrene	12	1
Picea	74	56
Pieris	0	1
Pinus	107	218
Pinuspollenites	46	1
Pistia	1	6
Pityostrobus	0	3
Planera	2	0
Platananthus	1	0
Platanites	1	0
Platanophyllum	15	0
Platanus	122	18
Platycarya	74	9

Table S6: Summary of the Cenozoic plant fossil data set (continued).

Genus	No. occurrences	
	NA	EA
Podocarpidites	0	1
Pongamia	2	0
Populus	158	68
Posidocea	0	1
Posidonia	0	19
Potamogeton	14	174
Protophyllum	2	0
Prunites	4	0
Prunus	42	47
Pseudosalix	1	0
Pterocarya	32	90
Pterospermites	6	0
Pyrenacantha	1	0
Quercus	175	106
Quereuxia	14	0
Rhamnidium	5	0
Rhamnites	14	0
Rhamnus	32	16
Rhododendron	2	6
Rhus	50	14
Rosa	22	14
Ruppia	2	4
Rutaspermum	0	15
Sabalites	1	0
Salix	147	75
Salvinia	35	110
Sapindus	8	7
Sapotispermum	0	1
Sassafras	38	7
Sequoia	29	121
Sloanea	10	0
Smilax	9	7
Sparganium	14	155
Spirodela	3	0
Stephania	0	1
Sterculia	3	0
Stillingia	9	0
Strongylodon	2	0
Tabernaemontana	1	0
Taxodium	82	145
Terminalia	4	0

Table S7: Summary of the Cenozoic plant fossil data set (continued).

Genus	No. occurrences	
	NA	EA
Ternstroemites	18	1
Thalassia	4	0
Thalassites	1	0
Thalassodendron	3	0
Thelypteris	14	0
Thouinopsis	9	0
Thuites	2	0
Tilia	24	35
Tinomiscoidea	1	2
Toddalia	0	33
Trapa	11	31
Triporopollenites	111	2
Trochodendroides	6	0
Ulmus	59	45
Vauquelinia	7	0
Viburnum	54	9
Vitis	38	181
Vouapa	1	0
Wardensheppeya	0	1
Weinmannia	1	0
Woodwardia	40	1
Zamites	9	0
Zannichellia	1	3
Zelkova	44	41
Zingiberopsis	63	0
Ziziphus	14	9
Zostera	0	2
